# Afterhyperpolarization Promotes the Firing of Mitral Cells through a Voltage-Dependent Modification of Action Potential Threshold

**DOI:** 10.1523/ENEURO.0401-21.2021

**Published:** 2022-04-01

**Authors:** Nicolas Fourcaud-Trocmé, Mickaël Zbili, Patricia Duchamp-Viret, Nicola Kuczewski

**Affiliations:** Lyon Neuroscience Research Center, CNRS UMR 5292 - INSERM U 1028 - Université Claude Bernard Lyon 1, Centre Hospitalier Le Vinatier - Bâtiment 462 - Neurocampus, 95 Boulevard Pinel, 69675 Bron Cedex, France

**Keywords:** AHP, burst firing, olfactory bulb, patch clamp, rat, sodium channel

## Abstract

In the olfactory bulb, mitral cells (MCs) display a spontaneous firing that is characterized by bursts of action potentials (APs) intermixed with silent periods. Intraburst firing frequency and duration are heterogeneous among MCs and increase with membrane depolarization. By using patch-clamp recording on rat slices, we dissected out the intrinsic properties responsible for this bursting activity. We showed that the threshold of AP generation dynamically changes as a function of the preceding trajectory of the membrane potential. In fact, the AP threshold became more negative when the membrane was hyperpolarized and had a recovery rate inversely proportional to the membrane repolarization rate. Such variations appeared to be produced by changes in the inactivation state of voltage-dependent Na^+^ channels. Thus, AP initiation was favored by hyperpolarizing events, such as negative membrane oscillations or inhibitory synaptic input. After the first AP, the following fast afterhyperpolarization (AHP) brought the threshold to more negative values and then promoted the emission of the following AP. This phenomenon was repeated for each AP of the burst making the fast AHP a regenerative mechanism that sustained the firing, AHP with larger amplitudes and faster repolarizations being associated with larger and higher-frequency bursts. Burst termination was found to be because of the development of a slow repolarization component of the AHP (slow AHP). Overall, the AHP characteristics appeared as a major determinant of the bursting properties.

## Significance Statement

Mitral cells (MCs) in the olfactory bulb are the main relay of olfactory information toward higher cortical areas, and their firing activity provides a substrate for olfactory information. The MC intrinsic dynamics generate a discharge of action potentials (APs) in burst patterns whose underlying mechanisms are not yet elucidated. Here, we show the importance of the AP afterhyperpolarization (AHP) in this process. The fast AHP component increases the availability of sodium channels, which facilitates the generation of burst discharge. In addition, the late manifestation of the slow AHP component returns the availability of sodium channels to their initial state and leads to the termination of a burst. Overall, we demonstrate that burst properties of MCs are determined by AHP characteristics.

## Introduction

The afterhyperpolarization (AHP) that follows the action potential (AP) is generally seen as an inhibitory mechanism that limits neuronal activity by promoting firing frequency adaptation and termination of the AP burst (Schwindt et al., 1988; Faber and Sah, 2007; Adelman et al., 2012; Reuveni and Barkai, 2018). The main mechanism underlying AHP is the activation of voltage- and calcium-dependent potassium currents. However, in some neuronal types, such as mitral cells (MCs), the main output neurons of the olfactory bulb, the synaptic transmission also contributes to the AHP shape (Duménieu et al., 2015). Differences in the activation–inactivation kinetics and calcium sensitivity of the different subtypes of potassium channels, underlying AHP, are responsible for a division of its course into three successive components, a fast, a medium, and a slow AHP, that differ in onset times, rise, and decay kinetics (Schwindt et al., 1988; Sah and Faber, 2002; Andrade et al., 2012). The relative contribution of each component evolves during the neuronal discharge, making the AHP shape dependent on the preceding neuronal activity (Duménieu et al., 2015). The inhibitory action of the AHP is generally attributed to the potassium channels that prevent excitatory currents to bring the membrane potential (*V*_m_) to the AP threshold (Rubin and Cleland, 2006). This vision may, however, lead to neglecting the possibility that AHP could potentially have a proexcitatory effect through the deinactivation of some voltage-dependent channels, such as calcium T-type and sodium channels (Deister et al., 2009; Cain and Snutch, 2010; Platkiewicz and Brette, 2011; Iyer et al., 2017). Such deinactivation would promote neuronal firing when the *V*_m_ moves back to the resting-state value.

MCs present a spontaneous firing activity that is characterized by AP clusters (i.e., bursts) interspaced by silent periods (Desmaisons et al., 1999). This activity is mainly because of intrinsic membrane properties, since it could be observed in pharmacologically isolated MCs, in olfactory bulb slices (Balu et al., 2004). The cellular mechanisms behind MC bursting activity remain to be elucidated. Balu and Strowbridge (2007) proposed that the burst termination was because of the buildup of the slow AHP during repetitive firing. A computational model refined this idea by proposing that burst termination is not because of the accumulation of classical AHP currents, but rather by the progressive deinactivation of potassium A-type current (*I*_A_) along the consecutive AHPs of the burst (Rubin and Cleland, 2006). However, the mechanisms that trigger the burst, maintain the sustained firing, and determine the number of APs or their frequency remain largely unknown.

Here we provide evidence that, through a dynamic change of AP threshold, the modification of *V*_m_ associated with the AHP act both as a burst regeneration process and as a burst termination mechanism. In this way, the AHP characteristics play a pivotal role in determining the firing properties of MCs.

## Materials and Methods

### Animals

Animal handling was conducted in accordance with the European Community Council Directive 86/609/EEC. Experiments were performed in postnatal day 30 (P30) to P42 male Long Evans rats (Janvier). The animals were maintained on normal light cycle and *ad libitum* access to water and food.

#### Slice preparation

Animals were anesthetized with an intraperitoneal injection of ketamine (50 mg/ml) and then decapitated. The head was quickly immersed in ice-cold (2–4°C) carbogenized artificial CSF (ACSF; composition: 125 mm NaCl, 4 mm KCl, 25 mm NaHCO_3_, 0.5 mm CaCl_2_, 1.25 mm NaH_2_PO_4_, 7 mm MgCl_2_, and 5.5 mm glucose, at pH 7.4) oxygenated with 95% O_2_ and 5% CO_2_. The osmolarity was adjusted to 320 mOsm with sucrose. The two olfactory bulbs were removed from the cranial cavity and cut into horizontal slices (400 μm thick) using a vibratome (model VT1000s, Leica Biosystems). Slices were then incubated in Gibb’s chamber at 30 ± 1°C in modified calcium and magnesium ACSF (2 mm CaCl_2_, 1 mm MgCl_2_).

#### Electrophysiological recordings

Slices were transferred into a recording chamber mounted on an upright microscope (Axioskop FS, Zeiss) and perfused with oxygenated ACSF (4 ml/min) at 30 ± 1°C. Neurons were visualized using a 40× objective and an Orca Flash 4.0 camera (Hamamatsu). Measurements were performed with a RK 400 amplifier (BioLogic). Data were acquired with a sampling frequency of 25 kHz on a PC-Pentium D computer using a 12 bit analog/digital to digital/analog converter (Digidata 1440A, Molecular Devices) and PClamp10 software (Molecular Devices). Patch-clamp recordings were achieved with borosilicate pipettes (outer diameter, 1.5 mm; inner diameter, 1.17 mm; Clark Electromedical Instruments), filled with the intracellular solution (131 mm K-gluconate, 10 mm HEPES, 1 mm EGTA, 1 mm MgCl_2_, 2 mm ATP-Na_2_, 0.3 mm GTP-Na_3_, and 10 mm phosphocreatine, at pH 7.3, 290 mOsm). In our experimental conditions, the equilibrium potential of chloride ions (ECl) was −110 mV, and that of potassium ions (Ek) was −92 mV. The calculated junction potential of 13 mV was corrected offline.

### Data analysis

#### Evoked activity

Experiments were performed in current clamp. A small steady membrane hyperpolarization was ensured by negative current injection to prevent spontaneous firing. For the experiments investigating the relationship between the level of hyperpolarization and the AP threshold, two successive APs were generated by two 3 ms depolarizing current steps applied at 6 s interval; the second step being preceded by membrane hyperpolarization varying in amplitude and duration. Some data were excluded from the analysis when the average resting potential, in the 500 ms preceding the depolarizing step, differed by >2 mV between the two evoked APs. In experiments investigating the relationship between the speed of repolarization–hyperpolarization and AP threshold, current ramps of variable slopes were applied. The AP threshold was calculated from the first AP generated during the ramp: it was defined as being the first point with a strict positive acceleration (second derivative of its *V*_m_) during the AP rising phase, before it reaches its maximum depolarizing rate.

#### Sodium current measurements

Experiments were performed in voltage clamp in the presence of 0.3 mm cadmium, 4 mm nickel, 10 mm tetraethylammonium, 10 mm 4-aminopyridine (4AP), 10 mm 2,3-dihydroxy-6-nitro-7-sulfonyl-benzo[*f*]quinoxaline, 5 mm 2-aminophosphonovalerate (APV), and 5 mm bicuculline.

#### Spontaneous activity

Spontaneous activity was recorded from cells at their resting membrane potential (17 cells with a resting potential at approximately −60 mV, and 15 cells at approximately −55mV; see Figs. 2, 3, 6–8). In some cells, we needed to inject a small steady depolarizing current to bring the MP from approximately −60 to approximately −55 mV to generate spontaneous-like activity (15 cells), while in two cells we needed to inject a small hyperpolarizing current to transform tonic firing to burst firing. We did not observe any qualitative differences among all groups of cells in our analyses; they are thus pooled in the description of results.

APs were detected each time the membrane potential crossed −23 mV (detection potential) from below, and the minimum interspike interval (ISI) was set at 1 ms. In one cell, the detection potential was set at −43 mV because of the low amplitude of the first AP in bursts.

Bursts were detected based on a time-interval threshold for the ISIs (tISIs), below which two occurring APs were assigned to the same burst. To achieve this, the tISI was first set at 90 ms for all analyzed MCs, and then adapted cell by cell in a recursive manner through the following procedure: we computed a new tISI as the median ISI of all detected bursts in a cell, plus four times their median absolute deviation (except for one cell: only 0.8 times its median absolute deviation; cell label 8 in the figures). If the new tISI was lower than the previous one, we used it again to detect bursts of the cell and restart over a new tISI computation and so on, until the new tISI was larger than the last one (final burst ISI thresholds: mean, 47 ms; SD, 19 ms; range, 13–90 ms). The reliability of the burst detection method was assessed by visual inspection of traces. In our analyses, we will always refer to the burst size as the number of APs within each burst.

**Figure 1.**
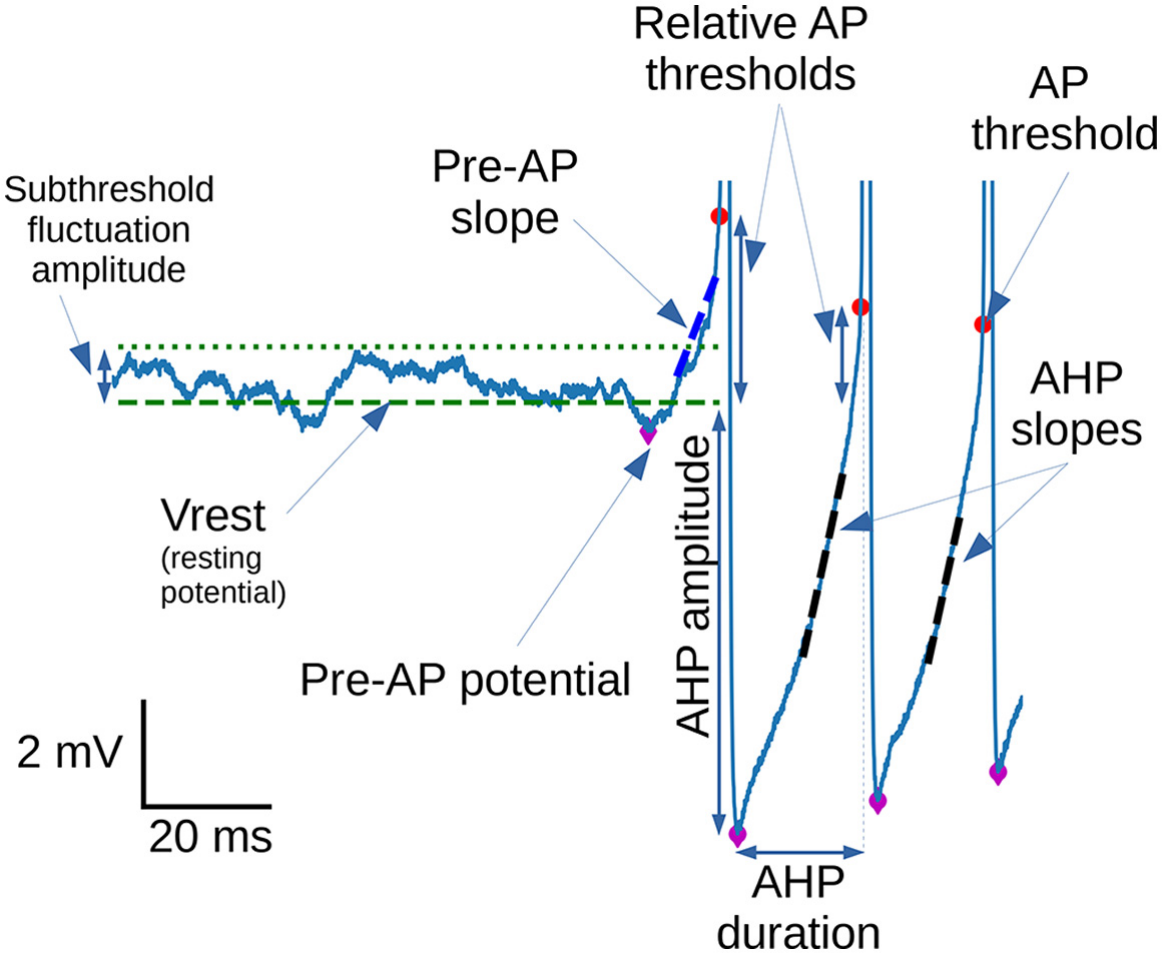
Schematic description of the main measurements performed on spontaneous bursts during the study. Red dots represent the thresholds of APs. Purple diamonds represent the most hyperpolarized value of *V*_m_ before one given spike (namely, “pre-AP potential” before the first AP and “AHP peak” before the other APs).

In the following, we describe how AP parameters or burst parameters used in this study were measured. An example of *V*_m_ trace with a schematic of most of the measures of interest is shown in Figure 1.

Because some recorded MCs showed slow fluctuations of subthreshold *V*_m_, the latter was computed before each burst. If the interval without an AP preceding a burst (interburst interval) lasted at least 200 ms, we defined the burst resting potential (*V*_rest_) as the median *V*_m_ during the interburst interval (excluding half of the tISI at the beginning and 5 ms at the end). We also defined subthreshold fluctuation amplitude as the maximum *V*_m_ on the same interval. If the interburst interval lasted <200 ms, *V*_rest_ and subthreshold fluctuation amplitudes were defined as the same as for the preceding burst. In some figures (see legends), traces are aligned on *V*_rest_, which was then set at 0 mV.

For each detected AP, we computed its voltage threshold (AP threshold) as the first point with a strictly positive acceleration (second derivative of *V*_m_) during the AP rising phase before *V*_m_ reaches its maximum positive acceleration rate. In this study, we often used the relative AP threshold defined as the difference between AP threshold and *V*_rest_.

The pre-AP potential was the first negative *V*_m_ peak that preceded the AP threshold. It was detected by stepping backward in time starting from the AP threshold by 1 ms steps and by stopping as soon as a *V*_m_ rebound of at least 0.4 mV (relative to the lowest *V*_m_ in the interval from current time step up to AP threshold time) was found. The pre-AP potential was defined as the lowest *V*_m_ between the rebound time and the AP threshold time.

The pre-AP slope was the slope of the *V*_m_ course preceding the first AP of the burst. When pre-AP potential occurred >5 ms before the AP threshold time, the pre-AP slope was obtained by a linear regression of the *V*_m_ course during the 5 ms before the AP threshold. When the pre-AP potential occurred <5 ms before AP threshold, the pre-AP slope was obtained by a linear regression of *V*_m_ course in a range of 20–80% from the pre-AP potential (0%) to the AP threshold (100%).

AHP amplitude was calculated by making the difference between the lowest *V*_m_ between two consecutive APs (or in the 300 ms following the AP, if the ISI was too long) and *V*_rest_. Note that, according to our convention, the AHP amplitudes are negative numbers.

The AHP slope was defined as the slope of a linear regression of *V*_m_ between the AHP peak and the next AP threshold, but restricted to a 20−60% *V*_m_ range starting from the AHP peak (0%) up to the previous AP threshold potential (100%). The *V*_m_ range for linear regression was indeed based on the preceding AP, to allow computation of the AHP slope following the last AP of a burst in the same way as the AHP slopes within the burst.

The AHP duration was defined as the period from the AHP peak to the next AP threshold time. The intraburst frequency was defined as the average of the inverse ISIs within the burst.

We noticed that, following a burst, a slow AHP component induced a slow repolarization of the *V*_m_ toward *V*_rest_. To quantitatively characterize this component for each MC, we selected all bursts with a following interburst interval of at least 500 ms. Electrophysiological traces were aligned on the AHP peak following the last AP, and a median trace was calculated. We then fitted this median trace from 50 to 500 ms after the AHP peak with a single exponential that gave the slow AHP time constant. Because of our choice of a minimum interval of 500 ms, some cells had no burst selected for median computation, the fit was thus possible in only 42 of the 49 cells used in this study.

#### Synaptically evoked activity

Experiments were performed in current clamp at MC resting membrane potential. Synaptic activity was produced by 0.1 ms electrical stimulation (8–10 mA) of the olfactory nerve layer that was produced by a bipolar electrode. Firing analysis was performed as for spontaneous activity.

### Threshold linear model

To predict the expected AP threshold following the last burst AP, for each cell, we fitted a model of intraburst AP threshold as a linear combination of AHP amplitude, AHP slope, AHP duration, and *V*_rest_ using an ordinary least squares regression method. Note that we also tested models taking into account interactions between these parameters, but the increase in fit reliability (based on the Bayesian information criterion) was negligible and did not justify taking these interactions into account.

Once a model was fitted for a given cell, we could predict the expected AP threshold following the last AP of each burst as a function of time elapsed after the last AHP maximum time (which gave the AHP duration parameter, with other model parameters being constant).

### Burst size and burst frequency linear models

To quantify the dependence of burst size and burst frequency on fast and slow AHP parameters, we pooled data from all cells and fitted models of burst size and burst frequency as a linear combination of *V*_rest_, AHP amplitude, and AHP slope (both measured during the first AHP within the burst) and their interactions. Linear models were fitted using an ordinary least squares regression method. Two additional models, including the slow AHP time constant (measured once for each cell as stated above), were fitted and statistically compared with the previous models with type I ANOVAs. Only bursts of more than three APs were included in this analysis. Normality and heteroscedasticity of residuals were checked visually and were required to transform the endogenous variables before model fitting as follows: log(log(burst size)) and log(burst frequency).

### Neuron computational model

A single compartment model was simulated with NEURON 7.8. All simulations were run with 100-μs time steps. The nominal temperature was 30°C. The voltage dependence of activation and inactivation of Hodgkin–Huxley-based conductance models were taken from the study by Hu et al. (2009) for Na_v_ and delayed rectifier potassium channels (KDR), and from the study by Rubin and Cleland (2006) for *I*_A_. The equilibrium potentials for Na^+^, K^+^, and passive channels were set to +90, −91, and −28.878 mV, respectively. We began by constructing a model with the following conductance densities: 0.02, 0.0002, 0.003, and 3.33 × 10^−5^ S/cm2 for Na_v_, KDR, *I*_A_, and passive channels, respectively. This model presented a resting membrane potential of −60 mV without holding current injection. In all the other model configurations, we injected a holding current during the simulation to maintain the resting membrane potential at −60 mV.

No *I*_A_ conductance was implemented in the model (see Fig. 5). The conductance density of Na_v_ was set to 0.02, 0.005, or 0.0025 S/cm^2^. The holding current was set to −0.826, −29.49, and −29.26 pA, respectively. Spikes were induced by 3 ms positive current steps of 400 pA. Hyperpolarizations before APs were induced by 50 ms negative current steps whose amplitudes were set to obtain a pre-AP membrane potential from −60 to −70 mV. AP threshold was defined by the voltage point at which the first-time derivative (dV/dt) went >40 mV/ms. The curve of AP threshold versus pre-AP activatable Na_v_ conductance was constructed by varying Na_v_ conductance density from 0.04 to 0.0025 S/cm^2^, inducing one spike from resting membrane potential and measuring AP threshold. The values of pre-AP activatable Na_v_ conductance were obtained by multiplying the percentage of noninactivated Na_v_ conductance—just before the positive current step—by the total Na_v_ conductance density.

For the figure 9 simulations, the Na_v_ conductance density was set to 0.02 S/cm^2^. The *I*_A_ conductance was either directly taken from the study by Rubin and Cleland (2006) or modified to get the biophysics values closer to previously published *I*_A_ biophysics values (Amendola et al., 2012). The modifications were done on the inactivation of *I*_A_ conductance: modified *I*_A_ displayed a more depolarized half-inactivation (−90 mV instead of −110 mV), a larger slope of inactivation curve (0.1 mV^−1^ instead of 0.056 mV^−1^), and a shorter inactivation time constant (50 ms instead of 150 ms). The holding current to keep the resting membrane potential at −60 mV was −30.826 pA for no *I*_A_ condition, 0 pA for *I*_A_ condition, and −5.85 pA for *I*_A_ modified condition. Spikes were induced by 3 ms positive current steps of 1 nA. Trains of spikes were induced by trains of these current steps, at 40 Hz.

### Statistical analysis

In many cases, we computed correlations between the different parameters characterizing the burst dynamics. We generally computed and plotted the within-cell correlations. Summary plots (mainly presented as extended data) show, for each cell, the slope of the correlation (left part of the figure), the strength of the correlation (*R* of the linear regression; right part of the figure). Single-cell statistically significant correlations (*p* < 0.05, corrected for multiple comparisons with the Bonferroni–Holm methods) are shown in dark blue (for both the slope and strength of the correlation). Global population statistical analysis was performed on the slopes and correlation coefficients of individual cells using standard *t* tests, assessing that the population averages were different from 0. Whisker plots give minimum and maximal values (whiskers), second and third quartile (box), and outliers calculated as deviations >1.5|*|IQR (interquartile range) from the first and third quartiles (diamonds). If not stated differently in results, the text gives the mean ± 95% confidence interval (CI; defined as 1.96 × SEM). Boxes show the mean and 95% confidence interval of the mean. Other quantities of interest are effect sizes (ESs), correlation coefficients (*R*), *t* values and *p*-values from *t* tests on *R* values, and the number of cells used in the analyses (*N*). Note that *p*-values from *t* tests performed on slopes are only given in figures. Bayesian analysis was performed with JASP Team (2020) by using the default effect size prior (Cauchy scale = 0.707).

### Exclusion criteria

Twenty-three spontaneously active MCs were excluded from the analysis based on the following reasons: 5 showed poor recording, 3 showed only continuous tonic activity, 11 showed too few bursts (<4 bursts), 2 had too high membrane resistance to be identified as MCs (>500 MΩ), and 2 presented burst intervals too short to compute resting potential (< 200 ms).

### Software

All analyses were performed with custom Python 2.7 scripts, using the statistical or curve-fitting functions from Scipy 1.2.2, and the multiple comparison functions or multiple regression functions from StatsModels 0.9.0.

### Code accessibility

Computational neuron model has been run with the software NEURON on a Linux personal computer. The corresponding code is freely available at the following address: https://osf.io/9bgxp/.

### Data availability

All raw electrophysiological traces, scripts for trace analyses, data analyses, and models scripts are available at Open Science Framework (https://osf.io/s2dbw/).

## Results

### Heterogeneity of spontaneous firing activity between different MCs

**Figure 2.**
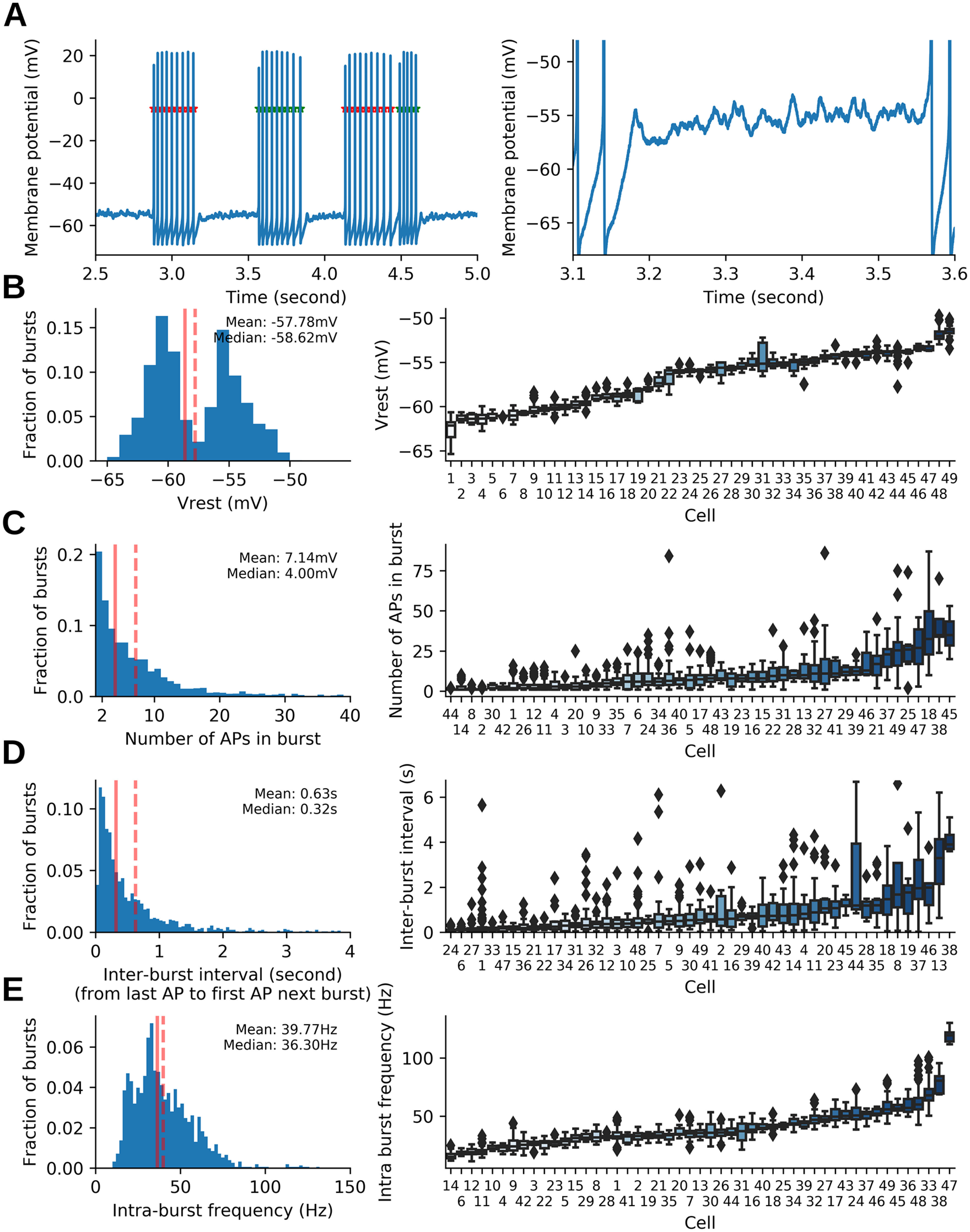
Burst properties of MCs. ***A***, Left, Example of spontaneous recording in MCs showing typical bursts of APs. APs belonging to the same burst are alternatively identified by red and green stars. Right, Enlargement of the trace in ***A***, left, showing *V*_m_ dynamics with fast oscillations, which were typical during interburst periods. ***B***, Distribution of *V*_m_ values preceding bursts (*V*_rest_). Left, Distribution for all detected bursts. Right, Whisker plot per cell showing intercell variability. ***C***, Distribution of burst sizes (number of APs in bursts, left and right: same representation as in ***B***). ***D***, Distribution of interburst intervals (same representation as in ***B***). ***E***, Distribution of intraburst frequencies (left and right: same representation as in ***B***). ***B–E***, Left panels, Continuous and dashed vertical lines materialize the median and mean values, respectively. ***B–E***, Right panels, diamonds correspond to outliers, calculated as deviations >1.5|*|IQR from the first and third quartiles.

Whole-cell recordings were performed on 72 spontaneously active MCs in olfactory bulb slices obtained from 21 rats between the ages of 30 and 42 d. Among recorded cells, 23 were excluded from the analysis based on criteria detailed in Materials and Methods. As previously shown (*C*hen and Shepherd, 1997; Desmaisons et al., 1999; Balu et al., 2004), firing activity was characterized by clusters of APs, henceforth denominated bursts, separated by silent periods presenting subthreshold membrane oscillations (Fig. 2*A*). A total of 1532 bursts (with at least 2 APs) and 386 isolated APs were analyzed. Burst properties such as the number of APs, the membrane potential at which bursts occurred, interburst frequency, and intraburst frequency were heterogeneous (Fig. 2*B–E*, left). Such heterogeneity could be partly because of specific differences among the recorded MCs (Fig. 2*B–E*, right), in which population intrinsic biophysical diversity is shown (Padmanabhan and Urban, 2010), but also because of the difference in the average holding potential (i.e., *V*_rest_) between the different MCs. In fact, more depolarized MCs presented higher intraburst frequency (*R* = 0.58, Wald test; *p* < 0.001; *N* = 49; data not shown) and larger burst size (i.e., larger number of APs in burst; *R* = 0.38, Wald test; *p* = 0.007; *N* = 49, data not shown). Burst size and intraburst frequency (*R* = 0.56, Wald test; *p* < 0.001; *N* = 49, data not shown) were also positively correlated. It should be noted that the *V*_rest_ distribution is bimodal (see Materials and Methods), but because such a bimodality was not expressed in other burst parameters, all cells were pooled in subsequent analyses.

### Dynamic modulation of AP threshold at resting potential

**Figure 3.**
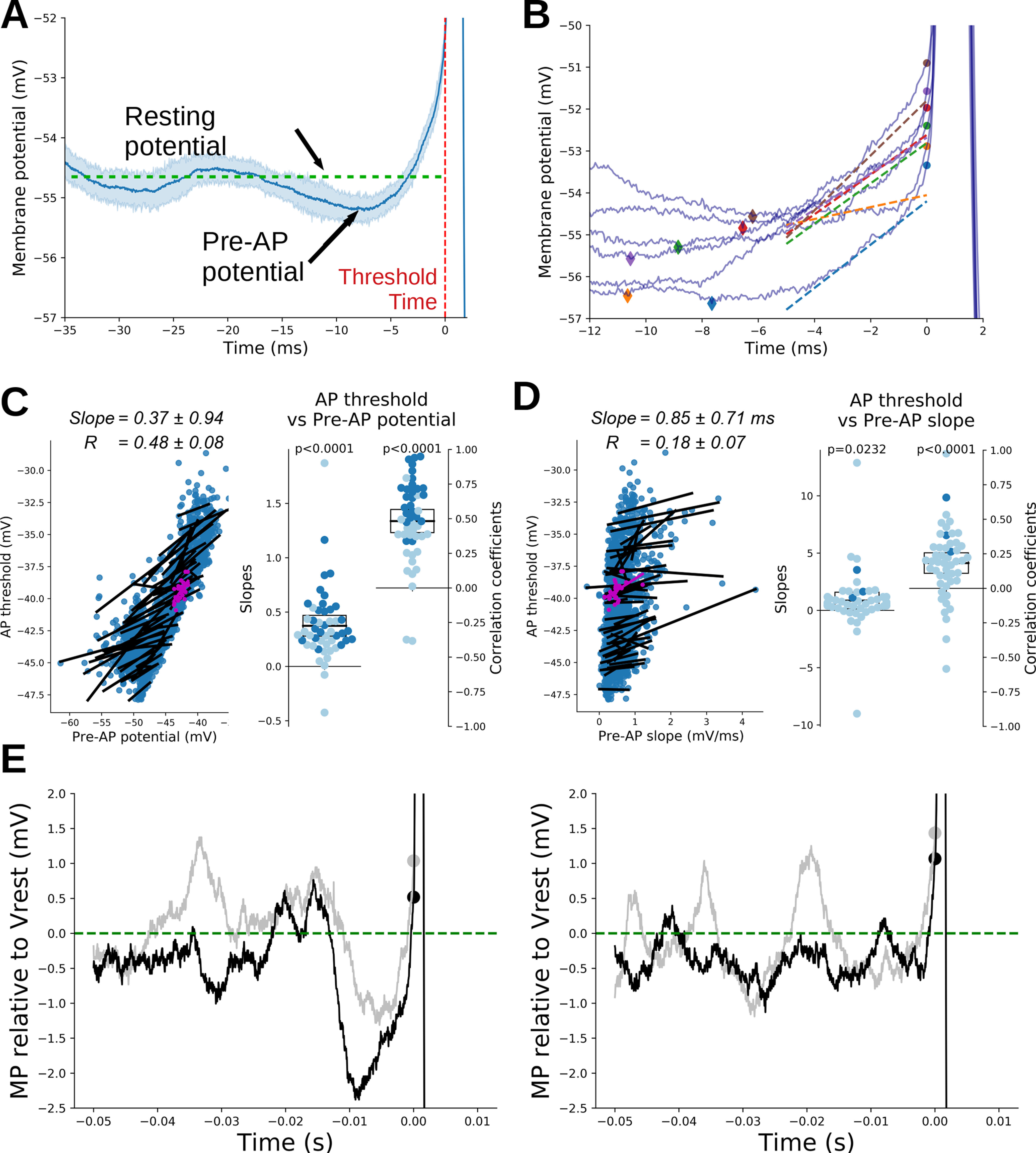
The threshold of the first AP of the burst is affected by the *V*_m_ trajectory before threshold. ***A***, Bursts were often initiated after a hyperpolarization phase of the *V*_m_ (pre-AP potential). Here, *V*_m_ traces preceding each burst of a single neuron (neuron #32) were averaged (blue line) along with their SD (shaded area). ***B***, Example of the variability of pre-AP slope and first AP threshold in the same cell (neuron #32). For each trace, a diamond marks the pre-AP potential, a round dot marks the first AP threshold, and a dashed line marks the pre-AP slope (lines are displayed in the time interval where *V*_m_ was fitted). Each color corresponds to one given trace. Note that a more negative threshold value corresponds to a more hyperpolarized pre-AP potential. See Materials and Methods and Figure 1 for the details of each measure. ***C***, AP threshold positively correlated with pre-AP potential. Left panels, Correlations between the pre-AP potential and first AP threshold. A linear fit was performed for each neuron (one dot per burst, the fit is shown as a black line). In purple are the measurements and fit of the neuron exemplified in ***A*** and ***B***. Average slope and average correlation across neurons are displayed above (mean ± SEM, *N* = 49 MCs). Right panels, Scatter plots of the slopes (left) and correlation coefficients (right) obtained from the linear fit done for each neuron in the left panel. Darker dots correspond to individual fits with *p* < 0.05 (Pearson correlation, corrected for multiple comparisons). Black boxes show the mean and its 95% confidence interval for each distribution. The significance of deviation from 0 of each distribution was further tested with a one-sample *t* test (*p*-values are displayed above each scatter plot; see main text and Materials and Methods for additional details). ***D***, Small positive correlation between the *V*_m_ slope preceding the first burst AP (pre-AP slope) and the first AP threshold. Data are presented as in ***C***. ***E***, Left, Examples (black and gray lines) of strong membrane hyperpolarization bringing the AP threshold (large dots) within the range of membrane fluctuations. Right, Examples of AP threshold that remained above membrane fluctuations. For comparison, traces were aligned on *V*_rest_ (set at 0 for the figure). All four examples come from the same recording (neuron #24).

In cortical neurons, the AP threshold is affected by the trajectory of *V*_m_ that precedes the AP. In particular, when the *V*_m_ is hyperpolarized or the rate of membrane depolarization (d*V*_m_/dt) preceding the AP is faster, a more negative AP threshold is observed (Henze and Buzsáki, 2001; Azouz and Gray, 2003; Li et al., 2014). Similarly, in MCs, the AP threshold is more negative when firing is preceded by *V*_m_ hyperpolarization induced by negative current injection (Balu et al., 2004). Interestingly, during spontaneous firing the initiation of a burst was always preceded by hyperpolarization of the *V*_m_ (Fig. 3*A*,*B*, example), which is in agreement with a previous report (Desmaisons et al., 1999). We therefore investigated whether the oscillatory activity, potentially associated with the spontaneous inhibitory transmission, was capable of producing a dynamic modification of AP threshold contributing to the firing initiation. In MCs, the threshold of the first AP of a burst was driven toward more negative values by a stronger hyperpolarization of *V*_m_ preceding the burst, which is hereafter called pre-AP potential (for its calculation, see Materials and Methods and Fig. 1). The average modification of AP threshold was −0.37 ± 0.94 mV for 1 mV pre-AP potential hyperpolarization (ES = 1.13, *R* = 0.48 ± 0.08, *t* = 11.6, *p* < 0.001, *N* = 49; Fig. 3*C*). We then analyzed the depolarization rate of *V*_m_ preceding the first AP, called here the pre-AP slope (Fig. 3*B*, examples; for its calculation, see Materials and Methods and Fig. 1). By contrast to what was reported for cortical neurons and predicted by theoretical models (Platkiewicz and Brette, 2011), in MCs, a larger pre-AP slope preceding the burst was associated with a more depolarized AP threshold (0.85 ± 0.71 mV, an increase in AP threshold per 1 mV/ms increase in pre-AP slope; ES = 0.34, *R* = 0.18 ± 0.07, *t* = 4.96, *p* < 0.0001, *N* = 49; Fig. 3*D*). This unexpected effect may be a consequence of the small positive covariation we measured between the pre-AP potential and the pre-AP slope [lower pre-AP slope for more hyperpolarized pre-AP potential; average slope, 0.04 ± 0.02 (mV/ms)/mV; ES = 0.52; *R* = 0.13 ± 0.07; *t* = 3.41; *p* = 0.0013; *N* = 49; data not shown]. According to the literature, this covariation should induce opposite effects on the threshold potential. Therefore, the pre-AP potential effect appeared to prevail over the pre-AP slope effect.

Since the AP threshold of MCs can dynamically shift depending on the recent history of *V*_m_, it is conceivable that the firing could be induced by hyperpolarizing events bringing the AP threshold below the median *V*_rest_ (for its calculation, see Materials and Methods and Fig. 1) or within the range of subthreshold *V*_m_ oscillations. This was indeed the case for 27% of recorded bursts (representing 92% of recorded MCs). Unsurprisingly, the level of hyperpolarization (pre-AP potential – *V*_rest_) preceding the burst was larger in these cases (Fig. 3*E*, left, example) than in bursts where the threshold of the first AP remained above the *V*_rest_ (Fig. 3*E*, right, example; hyperpolarization, −1.30 ± 0.17 vs −0.56 ± 0.07 mV; *t* = −9.48; *p* < 0.001; ES = 0.44; *N* = 513 and 1405; data not shown). Altogether, these data suggest that spontaneous firing in MCs could be triggered according to the following two modalities: (1) a membrane hyperpolarization produced by the oscillatory and/or inhibitory synaptic activity that brings the AP threshold below *V*_rest_ or within *V*_rest_ variability (Fig. 3*E*, left); and (2) a classical membrane depolarization above the *V*_rest_, eventually produced by the excitatory synaptic activity (Fig. 3*E*, right).

### Cellular mechanisms of AP threshold modification

**Figure 4.**
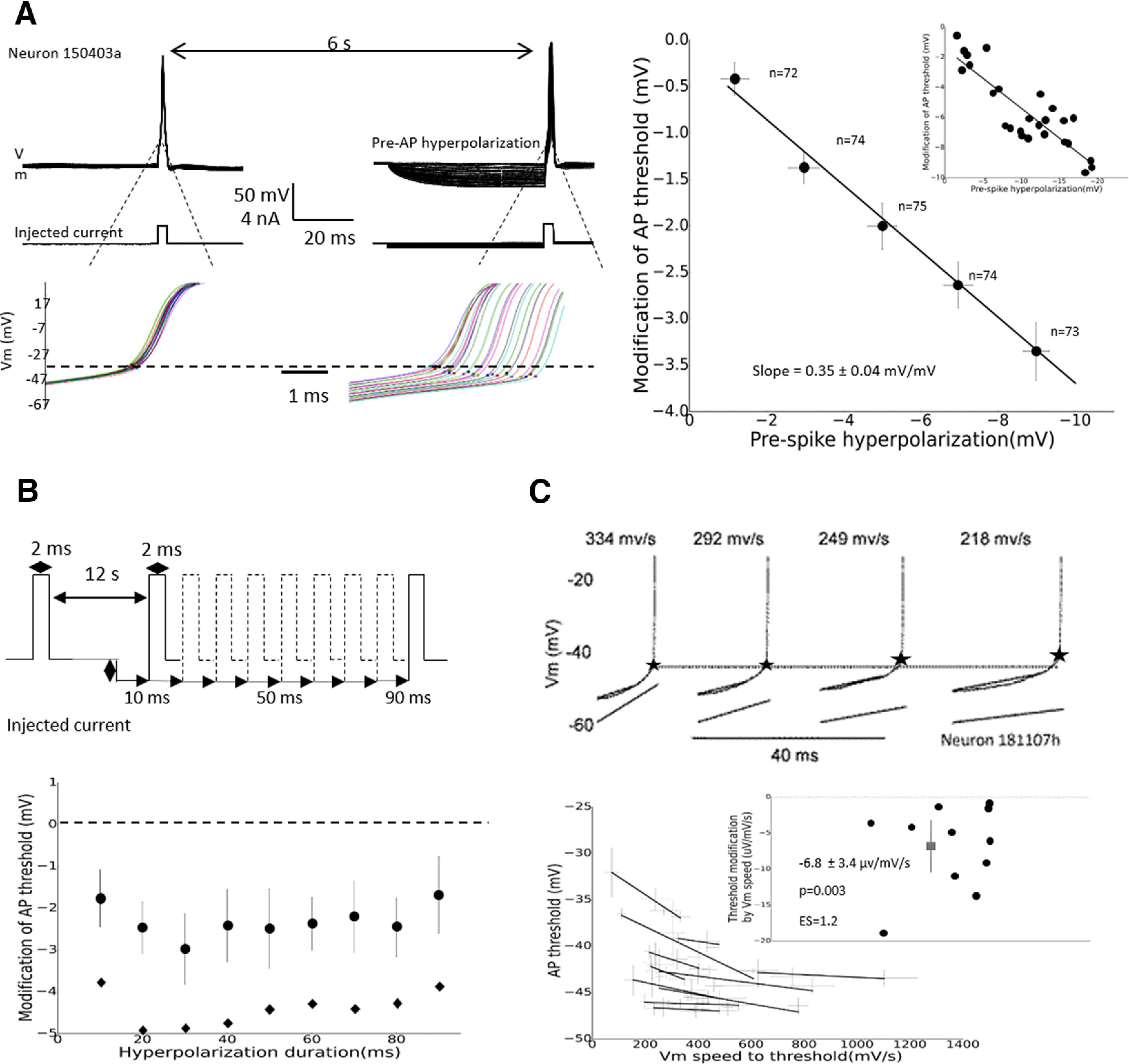
AP threshold of MCs decreased with membrane hyperpolarization and high depolarization speed. ***A***, AP threshold decreased proportionally with membrane hyperpolarization. Left, Experimental protocol and representative example of threshold modification by membrane hyperpolarization. Dots show AP thresholds, dashed line shows the threshold position in the absence of hyperpolarization. Right, Quantification of spike threshold modifications produced by membrane hyperpolarization. Inset, Analysis performed on neuron 150403a is depicted on the left. The number of cells is given above each point. ***B***, Spike threshold modification was not affected by the duration of membrane hyperpolarization. Top, Schematic representation of the experimental protocol. Bottom, Quantification of the effect (*N* = 16 MCs). Black circles, Average AP threshold modification; gray diamonds, average membrane hyperpolarization (same scale as threshold modification). ***C***, AP threshold decreased with the membrane depolarization rate. Top, Experimental protocol and representative examples of AP thresholds for different depolarization rates. Stars show the threshold position; lower lines materialize depolarization current ramps (only the last 10 ms preceding the threshold is shown). Bottom, Quantification of AP threshold as a function of membrane depolarization rate for 11 MCs. Inset, Quantification of AP threshold modification as a function of membrane depolarization rate for the 11 MCs. Horizontal and vertical bars represent 95% confidence interval.

The relationship between *V*_m_ and AP threshold was further investigated in the experiment depicted in Figure 4. Here MCs were slightly hyperpolarized with a steady current injection to prevent spontaneous firing, and two successive APs were evoked at 6 s intervals by short (3 ms) depolarizing current steps, with the second AP being preceded by a 50 ms hyperpolarizing current step (Fig. 4*A*, left). The comparison of AP thresholds between the two evoked APs showed that pre-AP membrane hyperpolarization produced a linear shift of AP threshold toward more hyperpolarized values (−0.32 ± 0.05 mV threshold shift per each millivolt of membrane hyperpolarization; Fig. 4*A*, right; *N* = 75). The same effect was obtained with pre-AP hyperpolarization duration varying over a range from 10 to 90 ms (Fig. 4*B*; *N* = 16). The contribution of the membrane depolarization rate to AP threshold was evaluated by comparing the effect of depolarizing ramps at different speeds (Fig. 4*C*, left). For the recorded MCs (*N*= 11), a faster depolarization rate leads to more negative AP thresholds (Fig. 4*C*, right). The slope of the linear regression obtained from AP threshold/depolarizing speed analysis showed a shift of AP threshold of −7 ± 3 μV for each millivolt per millisecond in membrane-depolarizing speed (Fig. 4*C*; right; ES = −1.2, *p* = 0.003). Therefore, similar to what was observed in cortical neurons (Henze and Buzsáki, 2001; Azouz and Gray, 2003; Li et al., 2014), AP threshold in MCs became more negative when *V*_m_ was depolarized with a fast depolarization rate. This result supports the interpretation that the opposite correlation between AP threshold and pre-AP slope during spontaneous firing (Fig. 3*D*) would be a consequence of a more hyperpolarized pre-AP potential when the pre-AP slope is lower.

**Figure 5.**
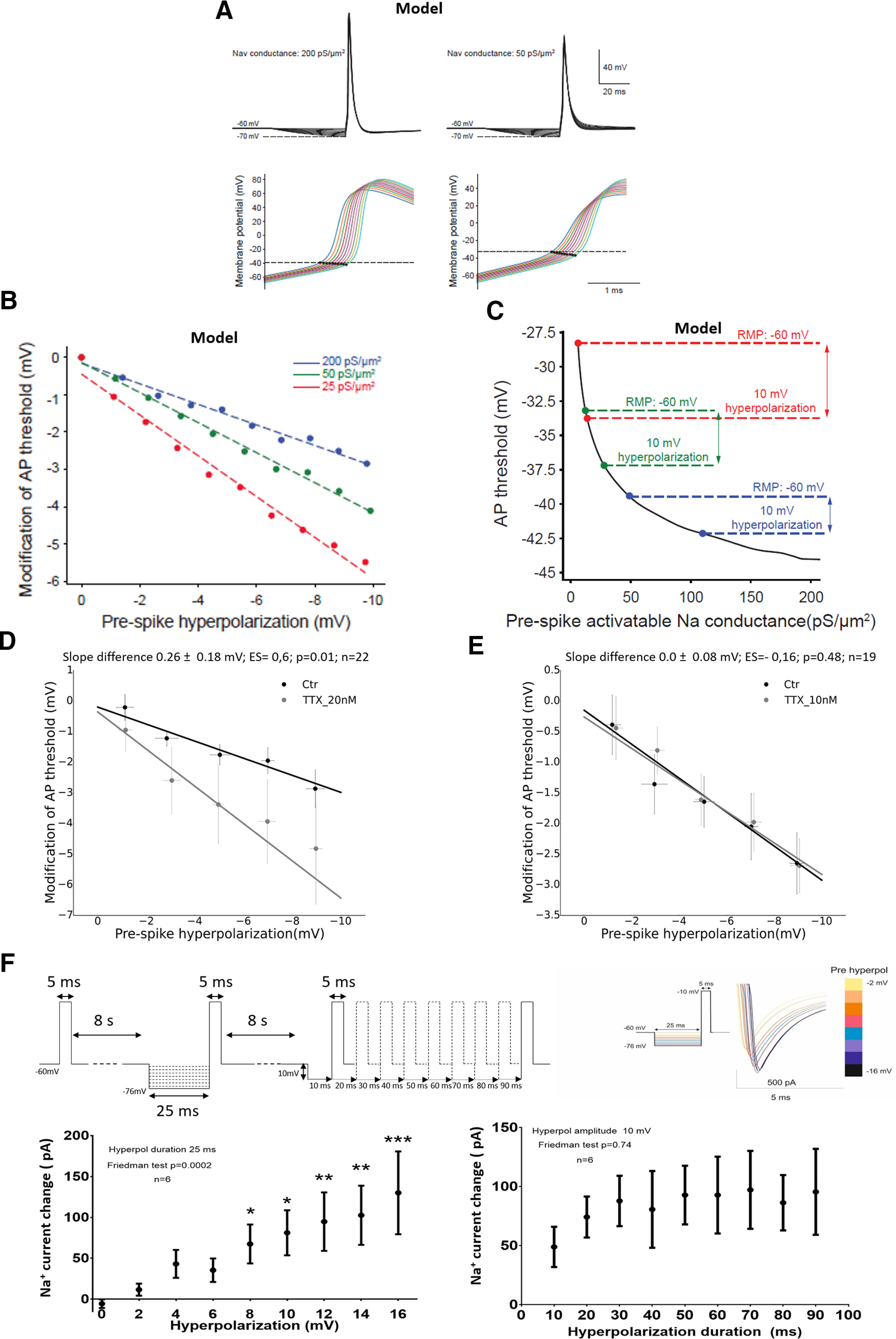
Sodium but not calcium channels participate in the modification of the AP threshold induced by membrane hyperpolarization. ***A***, Neuron model simulation showing the modification of AP threshold with membrane hyperpolarization for high Na_v_ density (200 pS/μm^2^) and low Na_v_ density (50 pS/μm^2^). Bottom panels are enlargements of top panels. AP thresholds are marked by black dots. ***B***, Quantification in the neuron model of the effect of membrane hyperpolarization on AP threshold for different Na_v_ channels densities. Note an amplification of the AP threshold shift induced by membrane hyperpolarization when the density of Na_v_ channels was reduced. ***C***, Curve depicting the modification of AP threshold in the neuron model as a function of the quantity of Na_v_ conductance activatable before the AP. Note that the same 10 mV membrane hyperpolarization produced a stronger modification of AP threshold when the density of Na_v_ channels was reduced (compare the following three conditions: 200, 50, and 25 pS/m^2^). ***D***, TTX at 20 nm amplified the AP threshold shift induced by membrane hyperpolarization. ***E***, TTX at 10 nm failed to amplify the AP threshold shift produced by membrane hyperpolarization. ***F***, Membrane hyperpolarization resulted in an amplification of Na^+^ current in MCs. Top left, Experimental protocol depicting the imposed modifications of membrane voltage. Top right, Representative of Na^+^ current following membrane hyperpolarization. Bottom left, Quantification of the modification induced by different levels of membrane hyperpolarization on Na^+^ current amplitude. Bottom right, Quantification of the modification produced, by different duration of membrane hyperpolarization, on Na^+^ current amplitude. *p < 0.02; **p < 0.01; ***p < 0.001, *post hoc* comparisons with the condition without hyperpolarization, Dunn’s test. Bars represent the 95% CI in ***A***, ***E***, and ***F***, and the SEM in ***G***. More details on pharmacological protocol, time stability of AP threshold, and effect of the voltage-dependent Ca^2+^ channel antagonist can be found in Extended Data Figure 5-1.

The most likely mechanism responsible for the modification of the threshold produced by membrane hyperpolarization is the recovery from inactivation of voltage-dependent channels implicated in AP generation; namely, the Na^+^ and T-type Ca^2+^ channels. To test this assumption, we used the same experimental procedure as described in Figure 4*A*, combined with a pharmacological approach as well as a computational neuron model. Since the pharmacological compounds were applied at different time periods, we first assessed that the effect of membrane hyperpolarization on AP threshold effect was stable over time by testing it 5 and 10 min after the beginning of the recording (Extended Data Fig. 5-1*C*,*D*). To investigate the effect of the recovery from inactivation of Na_v_ channels on AP threshold decrease, we first used a simple computational neuron model containing only one Na_v_ channel type and one K_v_ delayed-rectifier channel type (see Materials and Methods for details). This model closely mimicked the shift of AP threshold produced by the membrane potential hyperpolarization (Fig. 5*A*, left and 5*B*, blue). Thus, the shift could be attributable to the recovery from inactivation of Na_v_ channels produced by the *V*_m_ hyperpolarization, leading to a hyperpolarization of AP threshold. To observe the effect of the partial blockade of Na_v_ channels, we decreased the density of Na_v_ channels to 50 and 25 pS/μm^2^. As expected, decreasing the density of Na_v_ channels in the model, led to a shift of AP threshold toward more positive values (Fig. 5*A*, right). More interestingly, under reduced Na_v_ condition (50 and 25 pS/μm^2^), the hyperpolarization of membrane potential resulted in a larger decrease of AP threshold than in the control condition (Fig. 5*B*; note that the slope of the curve hyperpolarization/threshold shift became larger when the Na_v_ channels density is decreased). This can be explained by taking into account that the AP threshold is not linearly correlated with the quantity of activatable Na_v_ channels. Figure 5*C* shows the curve of the AP threshold versus the quantity of activatable Na_v_ conductance, just before the AP occurrence. On this curve, we plotted the following: the quantity of Na_v_ conductance activatable at *V*_rest_ (−60 mV) or after 10 mV hyperpolarization (−70 mV) for the three conditions of Na_v_ channels density (200 pS/μm^2^ in red, 50 pS/μm^2^ in green, or 25 pS/μm^2^ in blue). We can see that the increase in the availability of Na_v_ channels, produced by *V*_m_ hyperpolarization, is associated with a greater decrease of AP threshold in reduced Na_v_ condition (50 or 25 pS/μm^2^) than in the control condition (200 pS/μm^2^). The prediction of the model was checked by applying low doses of the Na channel blocker TTX (10 or 20 nm). The availability of Na_v_ channel was reduced by TTX at these doses, as assessed by the positive shift of AP threshold (Extended Data Fig. 5-1*B*). In agreement with the model prediction, the effect of membrane hyperpolarization on AP threshold was amplified by TTX at 20 nm (Fig. 5*D*; difference between threshold/hyperpolarization slopes in control and TTX 20 nm: 0.26 ± 0.18 mV/mV; ES = 0.6; *p* = 0.01; *N* = 22). Interestingly, this effect was not observed with TTX at 10 nm (Fig. 5*E*; difference between threshold/hyperpolarization slopes in control and TTX 10 nm: −0.03 ± 0.18 mV/mV; ES = −0.16; *p* = 0.48; BF_10_ = 0.3; evidence of absence, *N* = 19). This result suggests that, at approximately −60 mV, the recovery from inactivation produced by the hyperpolarization would mainly involve the Na_v_ channel subtypes blocked by [TTX]>10 nM (see Discussion). The participation of T-type Ca^2+^ channels was also investigated by using the selective antagonist ML218 (5–10 μm). As shown in Extended Data Figure 5-1*E*, ML218 did not modify the shift of AP threshold induced by membrane hyperpolarization (difference between threshold/hyperpolarization slopes in control and ML218: −0.0 ± 0.1 mV/mV; ES = 0.1; *p* = 0.66; *N* = 21), indicating that this effect was not based on recovery from the inactivation of T-type Ca^2+^ channels. We finally determined whether, in MCs recorded close to *V*_rest_ (−60 mV), short hyperpolarization of *V*_m_ could produce a recovery from the inactivation of Na^+^ channels. To this end, we performed the experiment depicted in Figure 5*F*. Here, Na^+^ current was pharmacologically isolated (see Materials and Methods) and MCs were recorded in voltage-clamp configuration. Following the short membrane prehyperpolarization (25 ms), the amplitude of Na^+^ current generated by a 5 ms depolarization step to −10 mV, increased proportionally to the hyperpolarization level (*p* = 0.0002, *N* = 6, Friedman test). Moreover, the recovery from the inactivation of Na^+^ channels was independent of the duration of the hyperpolarization, in the 10–90 ms range (*p* = 0.74, *N* = 6, Friedman test). These results echoed the effects produced by membrane hyperpolarization on AP threshold that were depicted in Figure 4, *A* and *B*, further supporting the hypothesis that the threshold shift was based on the recovery from inactivation of Na^+^ channels.

10.1523/ENEURO.0401-21.2021.f5-1 Figure 5-1 Modification of MC AP threshold and hyperpolarization effects over time and after applications of Na^+^ and Ca^2+^ channels antagonists. ***A***, Timing of experiments during which AP threshold was measured after 5 and 10 min, in control conditions or after different pharmacological applications. ***B***, AP threshold was significantly increased by blockade of Na^+^ channels with TTX, but not by the antagonist of T-type Ca^2+^ channels (ML218; 5–10 μm). Note the spontaneous increase of threshold with time in the control condition, both at 5 and 10 min, with a small difference, possibly due to cell dialysis. The statistical analysis compared with Ctrt0 is depicted above the data points. Values are expressed as the modification of AP threshold compared with the control condition at time 0. ***C***, ***D***, The effect of membrane hyperpolarization on the AP threshold was stable with time. ***E***, The antagonist of T-type channels ML218 did not affect the modification of AP threshold produced by membrane hyperpolarization. Horizontal and vertical bars represent 95% confidence interval. Download Figure 5-1, TIF file.

### Spontaneous bursting activity was promoted by dynamic modification of AP threshold induced by AHP

**Figure 6.**
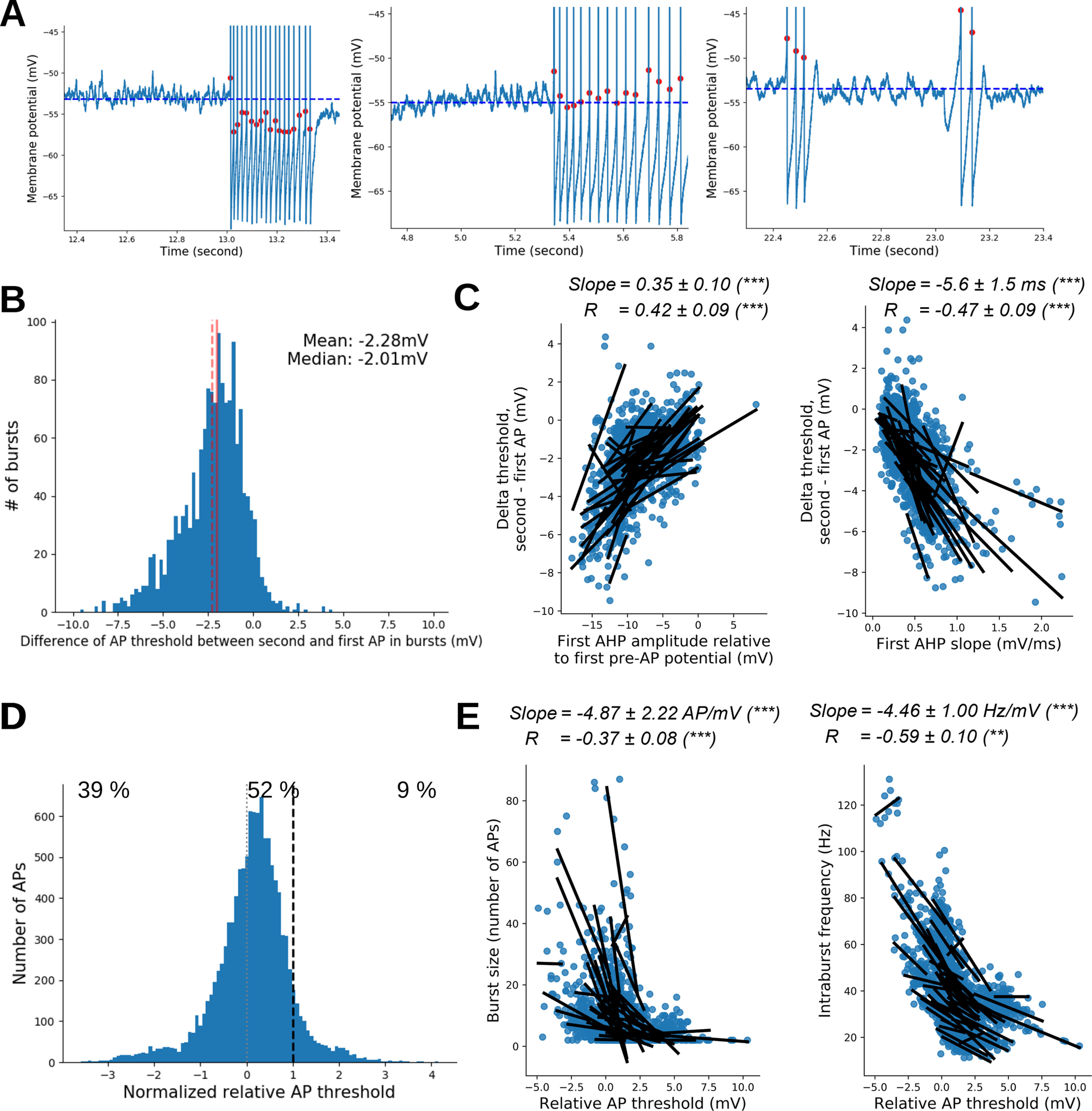
The AHP-induced hyperpolarization lowered the AP threshold within the bursts and determined the firing properties. ***A***, Examples illustrating the lowering of threshold for APs within the burst. The dashed line is *V*_rest_. Note that in the left and middle panels AP threshold is shifted below *V*_rest_ or inside *V*_m_ fluctuations, respectively. In right panel, the rebound of *V*_m_ observed after the AHP could potentially allow the burst generation despite the high AP threshold relative to *V*_rest_. ***B***, Histogram of differences in AP threshold between the second and the first AP of the bursts Continuous and dashed vertical lines materialize the mean and median values, respectively. ***C***, AHP properties determine the negative shift of AP threshold. Left, Larger AHP produced a larger negative shift of AP threshold. Positive correlation between the difference of the second and first AP threshold and the first AHP amplitude (computed here relative to the first pre-AP potential). Right, Faster AHP repolarization produced larger hyperpolarization of AP threshold. Negative correlation between the difference of the second and first AP threshold and the first AHP slope. ***D***, Distribution of the AP threshold relative to *V*_rest_ (first AP of each burst is excluded), normalized so that 1 (dashed line) corresponds to the maximum *V*_m_ reached during rest. ***E***, More negative values of relative AP thresholds were associated with bursts having a higher number of APs (left) and higher intraburst frequencies (right). Results in ***C*** and ***E*** are presented as in Figure 3*C*. See Extended Data Figures 6-1 and 6-2 for statistical details and individual-cell fit results. The relationship between AHP characteristics and firing properties can be found in Extended Data Figure 6-3. Examples of the characteristics of evoked firing can be found in Extended Data Figure 6-4.

We next investigated the impact of dynamic modifications of AP threshold on MC firing during the burst. As shown in Figure 6*A*, the AP thresholds within the bursts were naturally shifted to more negative potentials than those of the first AP (delta difference in threshold between the first and second AP in the burst = −2.28 ± 0.09 mV; *N* = 1532 bursts; ES = −1.28; *t* = −50.0; *p* < 0.001; Fig. 6*B*). The most likely explanation of the threshold shift within the burst is the membrane hyperpolarization induced by the preceding AHP. Indeed, the modification of AP threshold shift, calculated between the first and the second AP of the bursts, positively correlated with the AHP amplitude, measured here relative to the first pre-AP potential (−0.35 ± 0.10 mV of AP threshold shift for each millivolt of membrane hyperpolarization; Fig. 6*C*, left), as well as with the repolarization rate of the AHP (−5.6 ± 1.5 μV of AP threshold shift for each millivolt per second of modification of membrane depolarizing speed; Fig. 6*C*, right). These results suggested that both the AHP amplitude and repolarization rate accounted for the negative AP threshold shift within the burst. Interestingly, this effect, observed at the single-cell level, was also seen at the population level, by looking at average values per cell (Extended Data Fig. 6-1*C*,*D*). Once the first AP fired from resting potential, the AP threshold shift, induced by AHP, could act as a regenerative mechanism of firing, especially when it drove the threshold below the *V*_rest_ (Fig. 6*A*, left) or within the range of spontaneous membrane oscillations (Fig. 6*A*, middle). To precisely assess the position of AP thresholds relatively to subthreshold fluctuations, we normalized the thresholds of intraburst APs (i.e., all the APs except the first one) using a linear interpolation. We set *V*_rest_ at 0 and the maximum amplitude of the subthreshold fluctuations at 1. We called this measure the “normalized relative AP threshold.” In such conditions, a negative value for the normalized AP threshold meant that it was more hyperpolarized than *V*_rest_, a value between 0 and 1, which meant that it was within the range of *V*_m_ intrinsic subthreshold fluctuations and a value >1 meant that it was more depolarized than subthreshold fluctuations. As shown in Figure 6*D*, AP threshold was more negative than *V*_rest_ for 39% of intraburst APs: it remained within the range of subthreshold membrane oscillations for 52% and, above membrane oscillatory activity, for the remaining 9%. A clear heterogeneity of the normalized relative AP threshold was observed between MCs (Extended Data Fig. 6-2*A*). Interestingly the AP threshold shift relative to the *V*_rest_ (relative AP threshold), appeared to affect bursts properties. Stronger negative shifts were associated with burst having higher numbers of APs (Fig. 6*E*, left) and higher intraburst firing frequency (Fig. 6*E*, right). A similar correlation was observed between the average intraburst AP relative threshold in different MCs and their average burst sizes and intraburst frequency (Extended Data Fig. 6-2*C*).

10.1523/ENEURO.0401-21.2021.f6-1 Figure 6-1 Characteristics of the first AHP determined the negative shift of AP threshold between the first AP and second AP of the burst. ***A***, Distributions of slopes and correlation coefficients of the linear correlations performed in Figure 6*C*, left, of the main text (slope, 0.35 ± 0.10 mV/mV; ES = 1.03; *R*, 0.42 ± 0.09; *t* test, *t* = 9.34; *p* < 0.0001; *N* = 49). Darker dots correspond to individual fits with *p* < 0.05 (Pearson correlation, corrected for multiple comparisons). ***B***, Distributions of slopes and correlation coefficients of the linear correlations performed in Figure 6*C*, right, of the main text [slope, –5.6 ± 1.5 mV/(mV/ms); ES = –1.04; *R*, –0.47 ± 0.09; *t* test, *t* = –10.4; *p* < 0.0001; *N* = 49]. Darker dots correspond to individual fits with *p* < 0.05 (Pearson correlation, corrected for multiple comparisons). ***C***, Linear correlation between average first AHP amplitude and average negative shift of AP threshold (slope, 0.33 mV/mV; *R* = 0.57; Wald test, *p* < 0.001; *N* = 49). Each dot represents the average values for a given cell. ***D***, Linear correlation between average first AHP slope and average negative shift of AP threshold [slope, –3.4 mV/(mV/ms); *R* = –0.7; Wald test, *p* < 0.001; *N* = 49]. Each dot represents the average values for a given cell. Download Figure 6-1, TIF file.

10.1523/ENEURO.0401-21.2021.f6-2 Figure 6-2 Influence of intraburst relative AP threshold on burst size (number of APs) and intraburst AP frequency. ***A***, Same analysis as in Figure 6*D* of the main text but for each MC. ***B1***, Distributions of slopes and correlation coefficients of the linear correlations performed in Figure 6*E*, left, of the main manuscript (slope, –4.87 ± 2.22 AP/mV; ES = –0.62; *R*, –0.37 ± 0.08; *t* test, *t* = –9.56; *p* < 0.0001; *N* =49). Darker dots correspond to individual fits with p < 0.05 (Pearson correlation, corrected for multiple comparisons). ***B2***, Distributions of slopes and correlation coefficients of the linear correlations performed in Figure 6*E*, right, of the main manuscript (slope, –4.46 ± 1.00 Hz/mV; ES = –1.26; *R*, –0.59 ± 0.10; *t* test, *t* = –11.9; *p* < 0.0001; *N* = 49). Correlation between the intraburst frequency and relative AP threshold: individual-cell fit results from data shown in Figure 5*C*, right: average slope = – 4.46 ± 1.00 Hz/mV; ES = –1.26; average *R* = –0.59 ± 0.10; *t* test, *t* = –11.9; *p* < 0.0001; *N* = 49. Darker dots correspond to individual fits with p < 0.05 (Pearson correlation, corrected for multiple comparisons). ***C1***, Linear correlation between average relative AP threshold and average burst size (*R*, correlation coefficient; *p*, Wald test *p*-value; *N* = 49). Each dot represents the average values for a given cell. ***C2***, Linear correlation between average relative AP threshold and average intraburst frequency (*R*, correlation coefficient; *p*, Wald test *p*-value; *N* = 49). Each dot represents the average values for a given cell. Download Figure 6-2, TIF file.

10.1523/ENEURO.0401-21.2021.f6-3 Figure 6-3 AHP characteristics determined the firing properties of bursts, namely the burst size and intraburst AP frequency. ***A***, Larger AHP amplitudes were associated with longer bursts. ***A1***, Left, Correlations between AHP amplitude and burst size are found in a cell per cell analysis. Right, Distributions of slopes and correlation coefficients (slope, –2.98 ± 1.92 AP/mV; ES = –0.43; *R*, –0.13 ± 0.12; *t* test *t* = –2.26; *p* = 0.03; *N* = 49). Darker dots correspond to individual fits with *p* < 0.05 (Pearson correlation, corrected for multiple comparisons). ***A2***, Linear correlation between average AHP amplitude and average burst size (*R*, correlation coefficient; *p*, Wald test *p*-value; *N* = 49). Each dot represents the average values for a given cell. ***B***, Larger AHP amplitudes were associated with higher intraburst frequency. ***B1***, Left, Correlations between AHP amplitude and intraburst frequency is found in a cell per cell analysis. Right, Distributions of slopes and correlation coefficients (slope, –3.31 ± 1.36 Hz/mV; ES = –0.69; *R*, –0.33 ± 0.12; *t* test, *t* = –5.23; *p* < 0.0001; *N* = 49). Darker dots correspond to individual fits with *p* < 0.05 (Pearson correlation, corrected for multiple comparisons). ***B2***, Linear correlation between average AHP amplitude and average intraburst frequency (*R*, correlation coefficient; *p*, Wald test; *N* = 49). Each dot represents the average values for a given cell. ***C***, Faster AHP repolarizations (i.e., larger AHP slopes) were associated with longer bursts. ***C1***, Left, Correlations between AHP slope and burst size are found in a cell per cell analysis. Right, Distributions of slopes and correlation coefficients [slope, 38.3 ± 22.7 AP/(mV/ms); ES = 0.48; *p* = 0.002; *R* = 0.24 ± 0.08; *t* test, *t* = 5.79; *p* < 0.0001; *N* = 49). Darker dots correspond to individual fits with *p* < 0.05 (Pearson correlation, corrected for multiple comparisons). ***C2***, Linear correlation between average AHP slope average and burst size (*R*, correlation coefficient; *p*, Wald test *p*-value; *N* = 49). ***D***, Faster AHP repolarizations (i.e., larger AHP slopes) were associated with higher intraburst frequency. ***D1***, Left, Correlations between AHP slope and intraburst frequency are found in a cell per cell analysis. Right, Distributions of slopes and correlation coefficients [slope, 71.0 ± 9.0 Hz/(mV/ms); ES = 2.24; *R*, 0.76 ± 0.07; *t* test, *t* = 21.7; *p* < 0.0001; *N* = 49). Darker dots correspond to individual fits with *p* < 0.05 (Pearson correlation, corrected for multiple comparisons). ***D2***, Linear correlation between average AHP slope and average intraburst frequency (*R*, correlation coefficient; *p*, Wald test *p*-value; *N* = 49). Note that correlation coefficients were higher when the AHP slope was considered with respect to the AHP amplitude. Download Figure 6-3, TIF file.

10.1523/ENEURO.0401-21.2021.f6-4 Figure 6-4 Synaptically evoked bursts are similar to spontaneous bursts. Examples of a cell that displayed a clear threshold shift between the first two APs (top panels) and another that did not (bottom panels). For each cell, left panels illustrate side by side a burst evoked by olfactory nerve stimulation and a spontaneous burst. Black lines, An example of stimulation that failed to evoke a burst; red dots, represent AP thresholds. Right tables compare features of evoked and spontaneous bursts (for top cell: 3 evoked bursts and 21 spontaneous bursts; for bottom cell: 5 evoked bursts and 367 spontaneous bursts). Table values are displayed as the mean (SD). Download Figure 6-4, TIF file.

As expected from the relationship between the AHP and AP threshold shift, the intraburst firing properties correlated with AHP properties. Larger AHP amplitudes were associated with longer bursts and higher firing frequencies (Extended Data Fig. 6-3*A1*,*B1*). In addition, faster AHP repolarizations were associated with longer bursts and higher firing frequencies (Extended Data Fig. 6-3*C1*,*D1*). We also observed that the speed of repolarization of AHP more strongly impacted MC firing properties than AHP amplitude. A similar contribution of AHP to MC firing properties was observed when performing between-cell analyses (Extended Data Fig. 6-3*A2*,*B2*,*C2*,*D2*), suggesting that firing heterogeneity among the different MCs reported in Figure 1 are, at least partly, based on the heterogeneity of their AHP characteristics with a predominant role of the AHP slope.

By using electrical stimulation of the olfactory nerve, synaptically evoked firing was induced in two MCs. As shown in Extended Data Figure 6-4, the evoked firing-induced bursts in these cells had similar properties to spontaneous ones, except that subthreshold EPSP lasted longer than the AP duration. These preliminary results suggest that, once APs are generated, the bursting characteristics of MCs are determined by their intrinsic biophysical properties (i.e., the AHP), regardless of the modality of AP induction.

**Figure 7.**
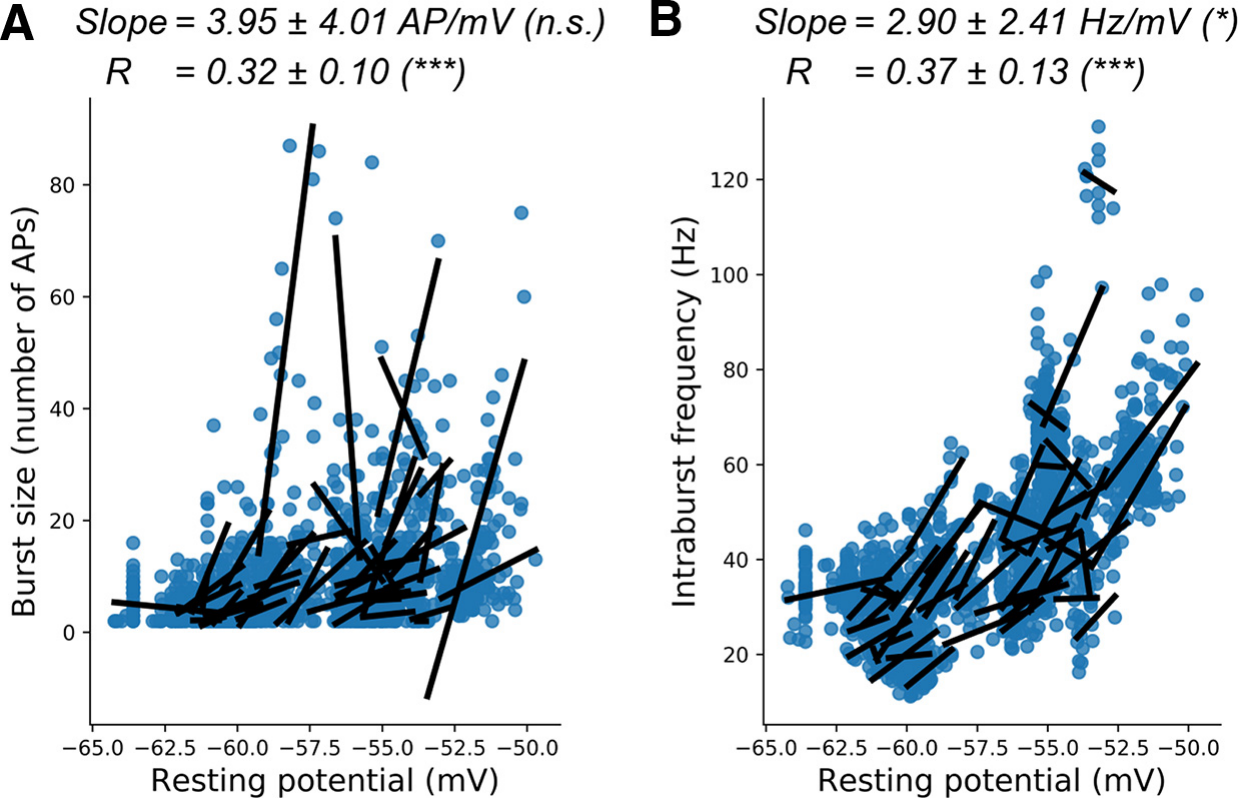
Influence of resting potential on burst size and frequency. ***A***, ***B***, Linear fits between burst firing properties and resting potential preceding the burst (*V*_rest_); results are presented as in Figure 3*C*. ***A***, Burst size (number of APs) increased with *V*_m_ depolarization. ***B***, Intraburst frequency increased with *V*_m_ depolarization. Statistical details and single-cell fit results are shown in Extended Data Figure 7-1, *A* and *B*.

So far, we provided evidence that the frequency and spike number of MC bursts were affected by the negative shift of AP threshold produced by the AHP, through the recovery from inactivation of Na^+^ channels. However, the burst properties depend also on the *V*_rest_. In fact, the frequency and spike number of MC bursts were increased when *V*_rest_ was depolarized (Fig. 7). This effect was likely because of the stronger negative shift of AP threshold within the burst when the *V*_m_ was depolarized (Extended Data Fig. 7-1*C*). The following three factors could support the latter effect: (1) an increase in AHP amplitude with *V*_m_ depolarization (Extended Data Fig. 7-1*D*), possibly because of the increase in K^+^ driving force with the membrane depolarization; (2) an increase in AHP repolarization speed of the AHP with *V*_m_ depolarization (Extended Data Fig. 7-1*E*); and (3) a stronger effect of Na_v_ channel recovery from inactivation, because of increased steady-state inactivation when the *V*_m_ was depolarized. Indeed, in the simple neuron computational model, a depolarization of the *V*_m_ led to a partial inactivation of Na_v_ channels, a reduction of the quantity of prespike activatable Na_v_ channels, and therefore a greater effect of *V*_m_ hyperpolarization on AP threshold shift (Extended Data Fig. 7-1*G*,*H*). The third factor is experimentally supported by the observation that the slope of the correlation between relative AP threshold and AHP amplitude was larger for depolarized MCs (i.e., for the same AHP amplitude, the AP threshold negative shift is larger for depolarized *V*_m_; Extended Data Fig. 7-1*F*). Overall, the three factors could cooperate to make the relative AP threshold more negative, thus making the bursts longer and faster for the more depolarized *V*_m_. All these effects, shown here at the cellular level, were also observed at the population level (cell-to-cell analysis; data not shown).

10.1523/ENEURO.0401-21.2021.f7-1 Figure 7-1 Influence of resting potential on burst size and intraburst frequency was linked to the modification of AP threshold, through changes in AHP characteristics and sodium channels inactivation rates. ***A–E***, Linear fits, slopes, and coefficients of the correlations between resting potential before burst and burst firing properties, AP threshold characteristics, or AHP characteristics. The first AP threshold in the burst is not taken into account. The results are presented as in Figure 3*C* (values are averaged per burst, and fits are done within each cell). ***A***, Burst size increased with *V*_m_ depolarization (slope, 3.95 ± 4.01 AP/mV; ES = 0.28; *R*, 0.32 ± 0.10; *t* test, *t* = 6.06; *p* < 0.0001; *N* = 49; left, identical to Fig. 7*A*). ***B***, Intraburst frequency increased with *V*_m_ depolarization (slope, 2.90 ± 2.41 Hz/mV; ES = 0.34; *R*, 0.37 ± 0.13; *t* test, *t* = 5.49, *p* < 0.0001; *N* = 49; left, identical to Fig. 7*B*). ***C***, Relative AP threshold became more negative with *V*_m_ depolarization (slope, –0.97 ± 0.29 mV/mV; ES = –0.94; *R*, –0.64 ± 0.10; *t* test, *t* = –12.3; *p* < 0.0001; *N* = 49). ***D***, AHP amplitude increased with *V*_m_ depolarization (slope, –0.59 ± 0.55 Hz/mV; ES = –0.30; *R*, –0.51 ± 0.14; *t* test, *t* = –7.32; *p* < 0.0001, *N* = 49). ***E***, AHP repolarization speed increased with *V*_m_ depolarization [slope, 0.036 ± 0.014 (mV/ms)/mV; ES = 0.73; average *R*, 0.33 ± 0.11; *t* test, *t* = 5.75; *p* < 0.0001; *N* = 49]. ***F***, Cells with a more depolarized *V*_rest_ showed a greater effect of pre-AP potential on AP threshold. In this panel, all the spikes of the burst are analyzed. For the first spike, pre-AP potential is the most hyperpolarized value of membrane potential preceding the burst while for the other APs, it corresponds to the negative peak of the preceding AHP (Fig. 1). Note that slopes of the correlation between relative AP threshold and pre-AP potential were bigger at more depolarized *V*_rest_ (i.e, the effect of a given hyperpolarization on AP threshold is bigger when resting potential is more depolarized). ***G***, Simulation of the effect of *V*_m_ hyperpolarization on AP threshold at different resting potential. It shows an amplification of the AP threshold shift produced by membrane hyperpolarization when the resting potential was more depolarized (note the change of the slope of the correlation between AP threshold modification and prespike hyperpolarization). ***H***, Modification of AP threshold as a function of the quantity of Na_v_ conductance available before the spike in the computational model. The dots represent the quantity of available Na_v_ conductance at resting potential or after 10 mV of hyperpolarization. Note that the same 10 mV membrane hyperpolarization produced a stronger modification of AP threshold when resting potential was more depolarized. Download Figure 7-1, TIF file.

### The evolution of the late slow component of the AHP contributed to the burst termination

**Figure 8.**
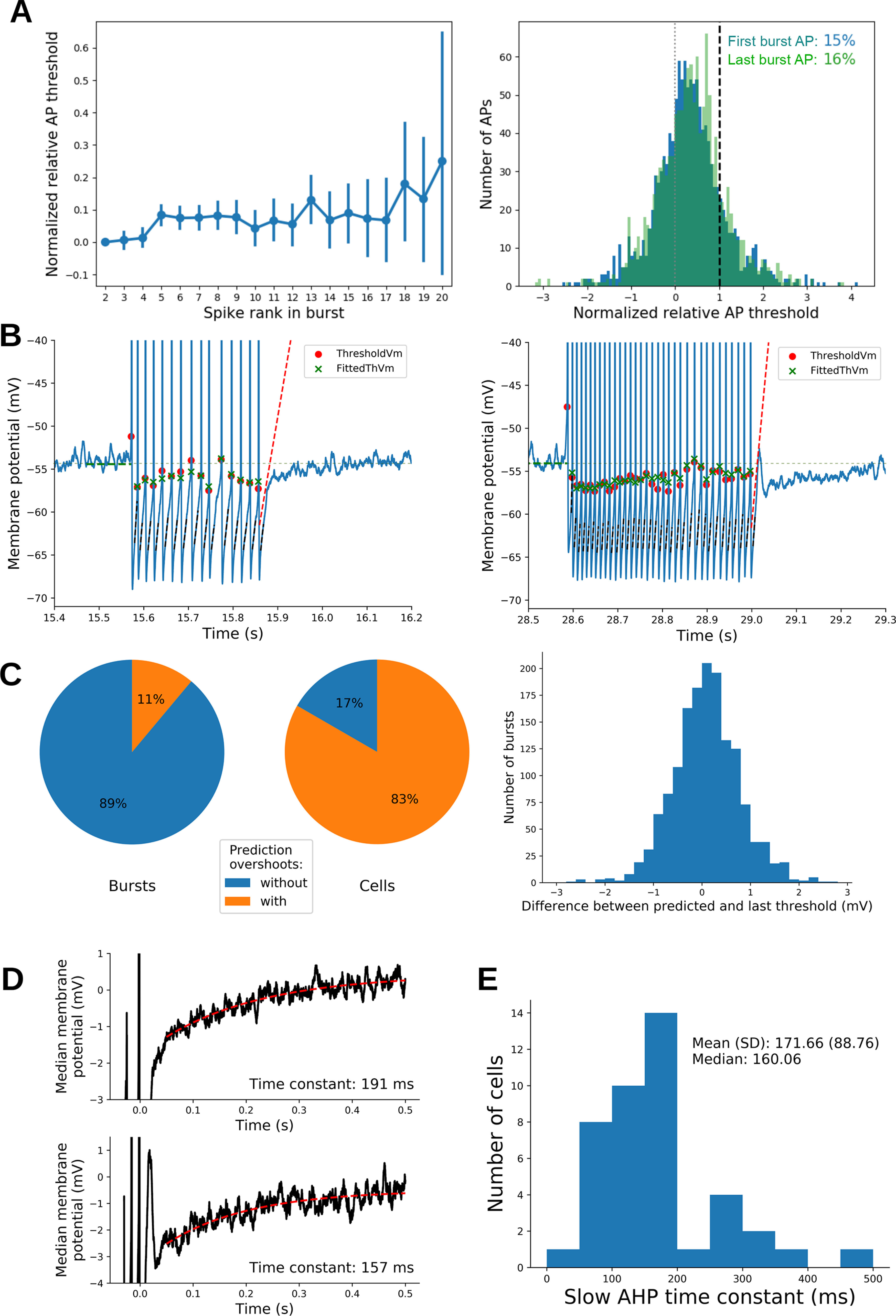
Bursts are terminated by the onset of a slow AHP component. ***A***, Left, Evolution along the burst of the normalized AP threshold (linear interpolation between 0 and 1, which were the resting potential and the maximum amplitude of subthreshold fluctuations, respectively). To compare between bursts, data were shifted and aligned at 0 for the second AP threshold. We observed a clear but small increase of normalized AP threshold during a burst. Right, Histograms of normalized AP thresholds for the second AP (blue) and last AP (green) in bursts (left panel). Percentiles give the proportion of data >1. ***B***, Examples of bursts with a detected AP threshold (red dots) and their model fits (green cross). Dashed red lines set the predicted threshold as a function of time elapsed after the beginning of the last AHP. We noted that bursts could be either followed by postburst rebound (right) or not (left). In both cases, the *V*_m_ stayed below the predicted AP threshold, accounting for the end of the burst, and a sudden decay of *V*_m_ repolarization rate was observed. See text and Materials and Methods for the model details. ***C***, As shown in ***B***, we could detect when the *V*_m_ goes above the predicted threshold (overshoot) without producing a further AP. Left, Pie chart shows the proportion of bursts with a predicted overshoot (pooled across cells). Middle, Pie chart shows the proportion of cells with at least one predicted overshoot among their bursts. Right, Distribution of the differences between predicted AP threshold (at the same ISI as the last burst ISI) and the threshold of the last AP in the burst. ***D***, Median traces of the 500 ms following the last AHP peak with exponential fits of the slow AHP component (computed from 50 to 500 ms following AHP peak). Top and bottom correspond to cells shown in ***B***, left and right, respectively. ***E***, Distribution of the time constant of the exponential fits shown in ***D*** for all 42 cells (see Materials and Methods for details). The evolution of the AHP characteristics along the burst can be found in Extended Data Figure 8-1.

A plausible mechanism that could account for the burst termination is the evolution of the AP threshold along the burst bringing this parameter above the *V*_rest_. However, although a small positive shift of the AP threshold was observed along the bursts (Fig. 8*A*, left; increase of normalized threshold: mean, 0.053 ± 0.027 mV; paired *t* test, *t* = 3.83; *p* < 0.001; *N* = 1276; ES = 0.11), the AP threshold remained largely below, or within, the range of *V*_m_ subthreshold oscillation (Fig. 8*A*, right). The small evolution of AP threshold probably reflected the small decrease of absolute AHP amplitude and the small slowing down of the AHP repolarization rate along the bursts (Extended Data Fig. 8-1). To further clarify the mechanisms of burst termination, we used a linear model (using *V*_rest_, AHP amplitude, AHP slope, and AHP duration as parameters; see Materials and Methods for details) that predicts the dynamics of the putative AP threshold after the last AP of the burst (Fig. 8*B*, red dashed lines, examples). This analysis showed that, for 89% of the bursts, the *V*_m_ that followed the last AP did not overcome the putative threshold (Fig. 8*C*, left). Such an effect cannot be explained by an overestimation of the threshold by the model since, at a time interval equal to the last interspike interval, the predicted threshold was similar to the threshold of the last AP of the burst (Fig. 8*C*, right; difference of potential between last AP threshold and predicted threshold, 0.09 mV; SD, 0.68 mV; *N* = 1532 bursts). Indeed, the failure of *V*_m_ to overcome the AP threshold appears to be because of the modification of the AHP, in which a late, slow repolarizing component develops following the early fast repolarizing component characterizing the intraburst AHP. This new AHP component could be fitted with a slow exponential (time constant: mean, 171.66 ms; SD, 88.76 ms; *N* = 42 MCs; Fig. 8*D*,*E*; Balu and Strowbridge, 2007, their Fig. 2C). This component kept the *V*_m_ more hyperpolarized than during the intraburst AHPs, preventing the *V*_m_ to reach the AP threshold. This result suggests a scenario where the AP threshold can only be reached during the early fast component of the AHP repolarization phase, with the onset of the late slow AHP repolarization preventing the *V*_m_ from reaching a threshold moving rapidly to preburst values.

10.1523/ENEURO.0401-21.2021.f8-1 Figure 8-1 Evolution of AHP parameters during bursts. ***A***, Same graphs as in Figure 8*A* (main text), but for AHP amplitude preceding the AP. Left, Distributions of AHP amplitude before the second AP and the last AP of the burst. There was a small decrease of AHP amplitude from the second to last spike (0.46 ± 0.04 mV; paired *t* test, *t* = 23.5; *p* < 0.001; *N* = 1276; ES = 0.65). Right, A small decrease of absolute AHP amplitude from the second to the third AP in burst, but after the third AP in burst the average AHP amplitude was constant. ***B***, Same graphs as in Figure 8*A* (main text), but for AHP slope preceding the AP. Left, Distributions of AHP slope before the second AP and the last AP of the burst. There was a decrease of AHP slope from second to last AP (–132 ± 9 mV/ms; paired *t* test, *t* = –27.8; *p* < 0.001; *N* = 1276; ES = –0.77). Right, The decrease of AHP slope occurs mainly during the first 10 APs in burst. Download Figure 8-1, TIF file.

**Figure 9.**
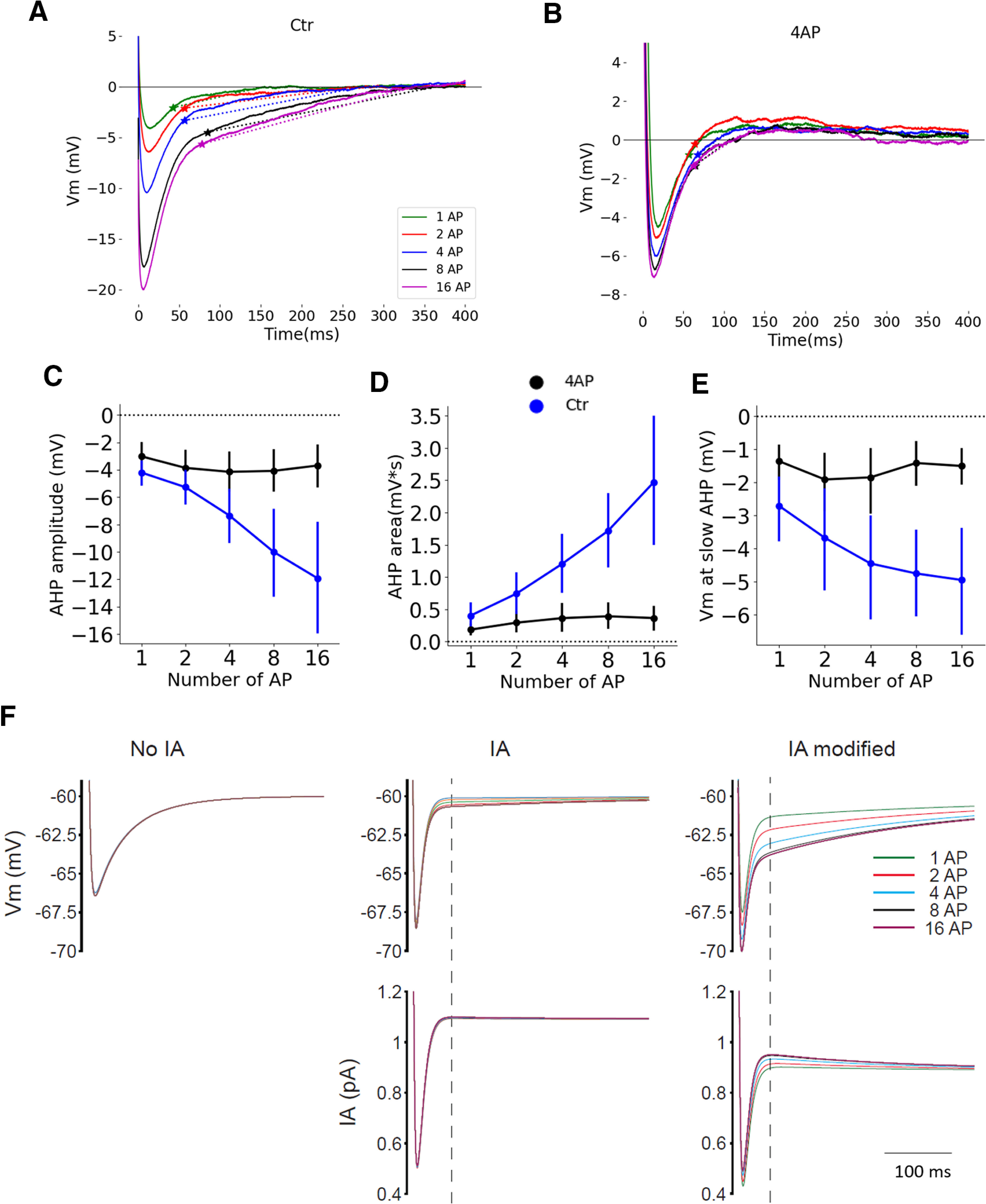
The AHP evolution with the number of APs was mainly because of 4AP-sensitive component. ***A***, Superposition of the last AHP of evoked trains comprising a different number of APs. Stars indicate the onset of the slow AHP component (i.e., at which the “*V*_m_ at slow AHP” was measured). ***B***, Same neuron as ***A*** but in the presence of 4AP (3 mm). ***C***, Modification of AHP, as a function of the number of APs in the trains, in the control condition and in the presence of 4AP. ***D***, Modification of the AHP area, as a function of the number of APs in the trains, in the control condition and in the presence of 4AP. ***E***, Modification of the *V*_m_ at which the slow component appeared, as a function of the number of APs in the bursts, in the control condition and in the presence of 4AP. ***F***, Neuron model showing the superposition of the last AHP at the end of evoked trains comprising different numbers of APs. Top Left, with only Na_v_ and KDR channels in the model, there was no evolution of the AHP with the number of APs. Top middle and right, Adding *I*_A_ current in the model reproduced the evolution of the slow AHP observed in experimental data. Bottom, Evolution of *I*_A_ current during the AHP. *I*_A_, channels biophysics from the study by Rubin and Cleland (2006); *I*_A_ modified, channels biophysics closer to those in the study by Amendola et al. (2012). Error bars represent 95% confidence intervals. *N* = 11 MCs.

Thus, the termination of burst discharge seems to be attributable to the onset of a slow component during the repolarizing phase of the AHP. To investigate how this slow component develops along the burst, we performed the experiment depicted in Figure 9. Here MCs were slightly hyperpolarized with a steady current injection, preventing spontaneous firing, while trains of APs were evoked by short (3 ms) depolarizing-current steps at 40 Hz. Five trains of 1, 2, 4, 8, and 16 APs, respectively, were generated, and the parameters of the last AHP were compared (Fig. 9*A*). As shown in Figure 9, *C* and *D*, the AHP amplitude and area increased with the number of APs (repeated-measures ANOVA, *p* < 0.001; *N* = 11). Interestingly, the slow AHP component appeared at more and more hyperpolarized *V*_m_ as the number of APs increased (repeated-measures ANOVA, *p* < 0.001; *N* = 11; Fig. 9*E*). This result suggests that, in spontaneous bursts, the probability that the fast AHP repolarizing component can overcome the AP threshold decreases along the AP sequence until it is no longer able to produce further APs. The slow late AHP component is reminiscent of the slow inactivating K^+^ current that was previously observed in MCs following membrane hyperpolarization and that was suggested to be produced by the recovery from inactivation of the *I*_A_ current (Balu and Strowbridge, 2007, their Fig. 5). The hypothesis that the slow AHP component is because of the deinactivation of the *I*_A_ current during fast AHP is further supported by a computational model of MCs showing that this current increases during the AP burst (Rubin and Cleland, 2006). Indeed, the application of 4AP (3 mm; Fig. 9*B*) prevented the evolution of AHP during the evoked AP trains (AHP area: 4AP effect, *p* = 0.005; interaction, *p* < 0.001; AHP amplitude: 4AP effect, *p* = 0.002; interaction, *p* < 0.001; Fig. 9*C*,*D*) as well as the negative shift of *V*_m_ at which the slow AHP component appears (4AP effect, *p* = 0.004; interaction, *p* < 0.001; Fig. 9*E*). Note that 4AP reduced both the early-fast and late-slow AHP. The hypothesis that the slow component involves the activation of *I*_A_ current is further supported by our neuron model (Fig. 9*F*). In fact, in the simple model involving only Na_v_ and KDR channels, there was no development of a slow AHP component when the number of APs increased (Fig. 9*F*, left, no *I*_A_). However, the implementation of *I*_A_ current to the model reproduced the development of a slow AHP with the number of APs, as observed in our recordings (Fig. 9*F*, *I*_A_ and *I*_A_ modified). The conductance of *I*_A_ was either directly taken from (Rubin and Cleland, 2006) or was modified, to get the biophysics closer to previously published *I*_A_ biophysics (Amendola et al., 2012; see Materials and Methods). Noteworthy is that the *I*_A_ current is minimal at the AHP peak and then increases again during the repolarization phase of the fast AHP, leading to the development of the slow AHP component (Fig. 9*F*, bottom panels). Altogether, these data suggest that, in MCs, the burst is stopped by the buildup of the 4AP-dependent, *I*_A_-like current that, by slowing down the AHP, brings the AP threshold to values that cannot be reached during *V*_m_ repolarization.

### The fast AHP features from the first spike of the burst and slow AHP dynamics are informative about intraburst frequency and burst size

Altogether, our experiments suggested that, in MCs, the burst frequency is determined by the fast AHP component while the burst termination (and thus the burst size) is determined by the development of the slow AHP during the burst. Therefore, we decided to observe whether the features of the fast and slow AHP, measured respectively after the first and the last AP of the burst, are good predictors of burst dynamics.

First, we only considered *V*_rest_ and fast AHP features. Pooling data from all cells (but only from bursts with at least three APs), we used linear models to predict burst size (number of APs) and within-burst frequency based on the following: *V*_rest_, fast AHP amplitude, and fast AHP depolarization rate measured after the first AP of each burst (see Materials and Methods for model details). The models showed that burst frequency clearly depends on all parameters and their interactions (*R*^2^ = 0.69; *F*_(10,1081)_ = 410.9; *p* < 0.0001; *N* = 1092 bursts). A similar but smaller effect was observed for burst size (*R*^2^ = 0.27; *F*_(10,1081)_ = 66.3; *p* < 0.0001; *N* = 1092 bursts). Thus, fast AHP dynamics following first AP is more informative of burst frequency than burst size.

Then, we also considered slow AHP features by introducing, in the linear models, the average time constant of the slow AHP (measured once in each cell; Fig. 8*E*). It should be noted that what we expected to be important in controlling the burst termination was the development of the slow AHP during the successive APs of the burst, but this could not be measured from the traces of spontaneous bursting activity. We therefore assumed that the development of the slow AHP correlated with its average time constant, which we could measure at the end of the burst. The addition of the mean time constant of the slow AHP to the linear model independent variables significantly improved their fitting performance, in particular in burst length (ANOVA comparisons of models with and without the time constant of the slow AHP as parameter: frequency: *F*_(4,1081)_ = 28.6, *p* < 0.0001; burst size: *F*_(4,1081)_ = 25.5, *p* < 0.0001; *R*^2^ of the new linear models and effect size (*f*^2^) between models: frequency: *R*^2^ = 0.72, *f*^2^ = 0.03; burst size: *R*^2^ = 0.33, *f*^2^ = 0.06).

Overall, the linear model analysis confirmed that the burst properties largely depend on the characteristics of the initial fast AHP and *V*_rest_, the slow AHP impacting the termination of the burst, and, thus, the burst size.

## Discussion

**Figure 10.**
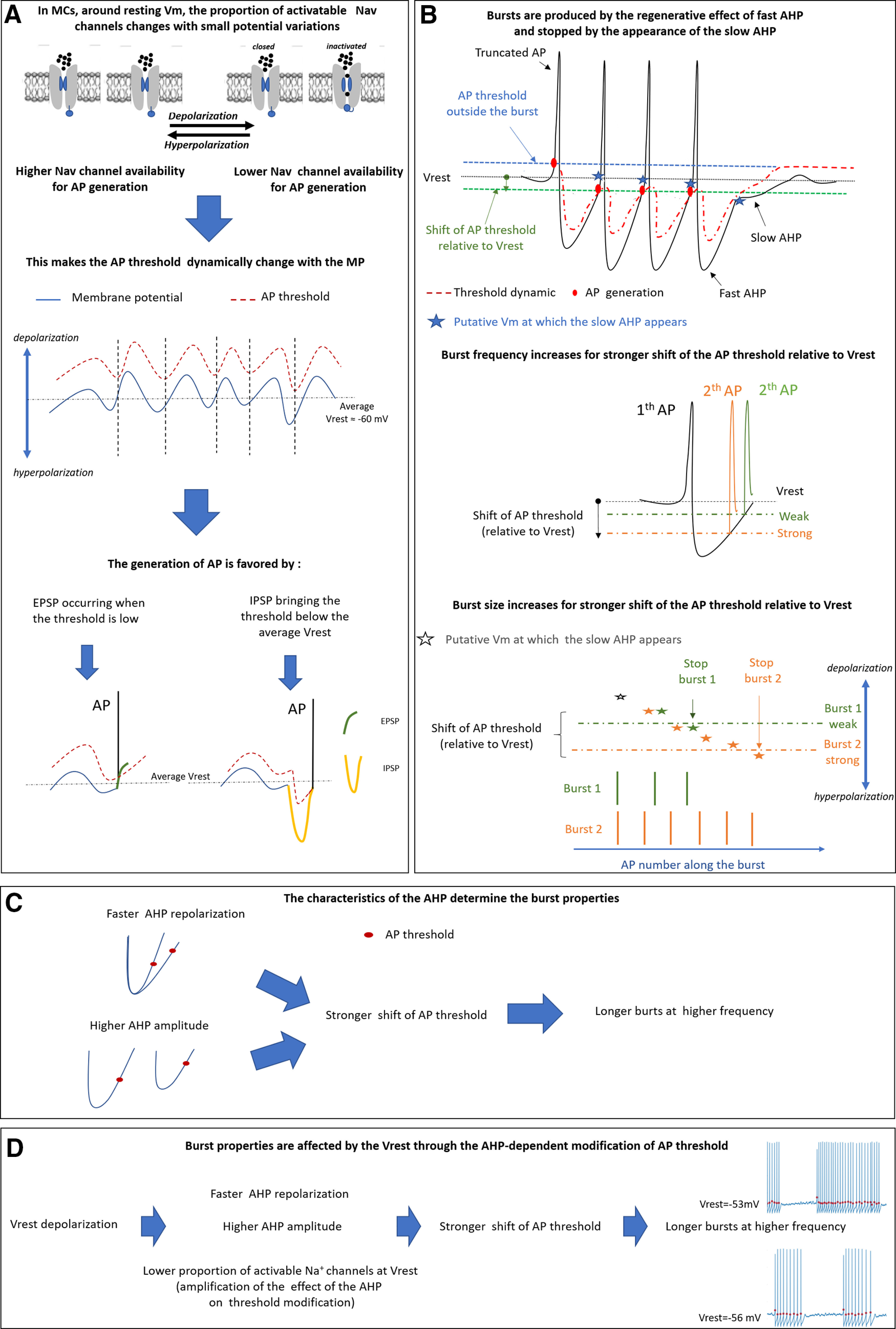
Model of the intrinsic mechanisms accounting for MC firing. ***A***, Transitions of Na_v_ channel between deinactivated and inactivated make the AP threshold dependent on *V*_m_ fluctuations. Because of the delayed deinactivation/inactivation kinetics, the threshold fluctuation (red dashed line) is slightly shifted compared with *V*_m_ fluctuations (blue line). The generation of the AP is favored by excitatory inputs occurring just after the negative phase of *V*_m_ fluctuations or when the repolarization phase of inhibitory inputs is rapid enough to overcome the shifted threshold before the latter goes back to the pre-AP values. ***B***, The fast AHP brings the AP threshold below the *V*_rest_, and its repolarization rate is rapid enough to overcome the modified threshold, acting in this way as a regenerative mechanism that sustains the burst. The slow components of the AHP develop gradually along the successive APs. When the *V*_m_ at which the slow component should appear is above the shift of AP threshold, the manifestation of the slow component is bypassed by the AP generation. Indeed, the slow AHP can manifest itself only at a *V*_m_ value more negative than the AP threshold shift. When this happens, the *V*_m_ repolarization during the slow component is not fast enough to overcome the AP threshold, which then returns to the preburst value, and the burst stops. For a stronger shift, the AP threshold is reached earlier, during the repolarization phase of the fast AHP, and the intraburst frequency is higher. For a stronger shift of AP threshold, a higher number of APs is necessary so that the slow AHP could manifest itself and, thus, the burst duration increases. ***C***, The threshold shift is larger when the fast AHP is larger and faster. In such conditions, the bursts are longer and have higher intraburst frequency. ***D***, The AP threshold shift is larger when the *V*_rest_ of the MC is more depolarized, because of modifications of AHP properties and partial inactivation of Na_v_ channels. As a consequence, *V*_m_ depolarization makes bursts longer and with higher intraburst frequency.

Our study provides new insights into the understanding of the intrinsic cellular mechanisms responsible for the genesis of firing activity in MCs. More precisely, we have shown that AHP plays a key role in this genesis since changes in its characteristics (duration, amplitude, kinetics) can both trigger and stop the burst generation while also determining the bursting properties. The experimental results presented in this report have been synthetized to build the toy model described in Figure 10. The firing of MC is triggered by a modification of the AP threshold that dynamically changes as a function of the *V*_m_ trajectory (Fig. 10*A*). Because of the relatively depolarized *V*_rest_ of MCs, a part of the voltage-dependent Na^+^ channels is inactivated. Hyperpolarization of *V*_m_ because of intrinsic oscillatory activity and/or synaptic inputs leads to Na_v_ channel deinactivation, bringing the AP threshold within *V*_m_ subthreshold fluctuations, thus facilitating the firing. Once the first AP is generated, the following fast AHP brings the threshold below the *V*_rest_, or within the *V*_rest_ noise, acting in this way as a regenerative mechanism that will produce the burst (Fig. 10*B*). The burst termination is ensured by a slow, 4AP-dependent AHP component that progressively develops along the consecutive APs and is hypothesized to involve the *I*_A_ current. This component slows down the AHP repolarization phase, and thus increases the inactivation of Na_v_^+^ channels and moves back the AP threshold to values that cannot be overcome by *V*_m_ repolarization. The intraburst properties (frequency, length) are determined by the magnitude of the modification of the AP threshold relative to the *V*_rest_. The larger the modifications of the AP threshold, the larger the bursts (in terms of the number of APs) and the higher the intraburst firing frequencies (Fig. 10*C*). Indeed, the firing frequency increases because the AP threshold is reached faster during the AHP repolarization phase, especially when the shift of the AP threshold toward hyperpolarizing values is associated with faster AHP repolarization. In addition to burst size increases, the AHP slow component needs more APs to manifest itself at *V*_m_, more negative than the AP threshold (Fig. 10*B*, middle and bottom panels). Finally, because the shift of the AP threshold during the burst is driven by the AHP, the burst properties are determined by the AHP features. In particular, the number of APs and firing frequency increase when the AHP amplitude and repolarization rate increase. The model also predicts the increases of burst length and intraburst frequency that we observed on membrane depolarization (Fig. 10*D*). In this condition, the AHP amplitude increases, possibly because of an increase of K^+^ driving force, and the AHP repolarization becomes faster, by an as yet unknown mechanism. Moreover, on membrane depolarization, the number of inactivated Na_v_ channels is augmented. As a consequence, the recovery from inactivation of Na_v_ channels produced by the AHP entails a larger shift of AP threshold relative to *V*_rest_. In fact, our neuron computational model showed that a decrease of the number of Na_v_ channels available entails an increase of the hyperpolarization-induced shift in AP threshold (Fig. 5*C*,*D*, Extended Data Fig. 7-1*G*,*H*).

Altogether, the heterogeneity of the firing properties observed among the different MCs would therefore be mainly because of differences in the shapes of their AHP components, as well as the recent history of *V*_m_ values.

However, our model does not predict the minority of intraburst APs for which the threshold was clearly above the *V*_rest_ (Fig. 6*A*, right). A mechanism that could account for these events is the rebound depolarization that was frequently observed at the end of the AHP. Obviously, when it could be detected, the rebound depolarization did not overcome the AP threshold, but a great variability in the rebound amplitudes was observed, supporting the above hypothesis. The post-AHP rebound depolarization is possibly because of the activation of persistent Na^+^ current (Balu and Strowbridge, 2007).

Not only depicting our own results, our model fits with others reported in the literature. As an example, the tufted cells of the olfactory bulb, which present an AHP with larger amplitude and faster repolarization rate than MCs, show a more sustained bursting activity; namely, longer and higher-frequency bursts (Burton and Urban, 2014). Similar covariation between AHP and burst properties have been also reported in MCs during postnatal development (Yu et al., 2015). Our model predicts that these covariations are the consequence of AHP-dependent modifications of the AP threshold shifts. In agreement with our hypothesis of the involvement of *I*_A_ current in burst termination, the application of 4AP has been reported to transform MC bursting activity into continuous firing activity (Balu et al., 2004). MCs present a dendritic recurrent synaptic transmission that is characterized by a glutamatergic autoexcitation (Aroniadou-Anderjaska et al., 1999; Friedman and Strowbridge, 2000; Salin et al., 2001) and a feedback inhibition that follows the activation of granular cells (Isaacson and Strowbridge, 1998; Schoppa et al., 1998). It has been shown that the main effect of recurrent synaptic transmission is to shape the AHP of MCs (Duménieu et al., 2015). In particular, the recurrent inhibition increases the amplitude of the AHP without affecting the medium or the late AHP, while the recurrent excitation reduces both the amplitude and the medium component of the AHP (Duménieu et al., 2015; Fig. 5*D*). Based on the present results, we can therefore propose that recurrent synaptic inhibition would favor long bursts at higher firing frequency; by contrast, the functional role of recurrent excitation is less predictable. Indeed, while the reduction of the AHP amplitude would favor short bursts at low frequency, we do not know whether, and to what extent, the reduction of the medium component would affect the velocity of the repolarization phase of the AHP; further analysis on this aspect is needed to elucidate the role of recurrent excitation on MC firing properties.

The interpretation of some of our experimental results requires further discussion: in particular, the contribution of *I*_A_ in the AHP course (Fig. 9) and the role of the recovery of the sodium channel in the modification of AP threshold (Fig. 5). As evidenced from Figure 9, an increase in AHP amplitude was observed when the number of evoked consecutive APs increased. This appeared as mainly based on the increase of the 4AP-dependent AHP component. Noteworthy was that during spontaneous bursts we did not observe any increase of AHP amplitude (Extended Data Fig. 8-1*A*). One possible explanation is that *I*_A_ currents develop earlier when the APs are evoked by experimental depolarizing steps, with the latter having a long duration (3 ms), relative to the AP half-width (∼0.7 ms). Moreover, the depolarizing step could mask the actual starting point of the AHP, giving the impression that the *I*_A_ component was already present at the beginning of the AHP. It is therefore plausible that during spontaneous firing activity *I*_A_ appears early enough to affect the medium/slow AHP, and therefore slows down the AHP repolarization during the burst, but too late to affect the amplitude of the AHP. The hypothesis of the involvement of the recovery from inactivation of Na_v_ in threshold modifications implies an increase of the effect of hyperpolarization when the global availability of Na_v_ channels is reduced (Fig. 5*B*,*C*). This was confirmed by the application of TTX at 20 nm but not when TTX was used at 10 nm. However, TTX 10 nm application is enough to reduce the Na_v_ channels availability as the AP threshold was shifted positively in this condition (Extended Data Fig. 5-1). The absence of the effect with TTX at 10 nm is probably not a consequence of sampling variability (i.e., a false-negative result) since Bayesian analysis supports an actual absence of the effect (BF_10_ less than one-third; Keysers et al., 2020). Moreover, when selecting only neurons for which both 10 and 20 nm TTX were applied, the 20 nm concentration reliably increased the effect of the *V*_m_ hyperpolarization on AP threshold while the 10 nm did not (data not shown). A plausible interpretation of these results is that the recovery from inactivation—produced by membrane hyperpolarization—may involve only those of the different subtypes of Na_v_ channels having a low sensitivity to TTX, thus not yet blocked at 10 nm. Further investigations are needed to confirm such a hypothesis, for example, by using selective antagonists of the different Na_v_ subtypes.

Last, our extensive analysis of the mechanisms that govern the discharge properties of MCs leads to the question of the functional significance and impact of these properties. The heterogeneity of the firing properties reported here confirmed previous reports of MC recordings *in vivo* and *in vitro* (Rinberg et al., 2006; Padmanabhan and Urban, 2010; Kollo et al., 2014; Leng et al., 2014). It has been proposed that such a diversity reduces the correlation of firing between different MCs to a correlated input, in this way increasing the information content of MC population activity (Padmanabhan and Urban, 2010). Moreover, computational simulations suggest that MC firing heterogeneity allows a more efficient and robust coding of stimulus information (Tripathy et al., 2013) and increases the synchronicity of MC firing when the correlation between the inputs is low, by possibly promoting encoding of odor combinations acting on different types of sensory receptors (Zhou et al., 2013). Our results show that the diversity among the population of MCs is largely determined by the heterogeneity of the AHP characteristics, which play a pivotal role in determining the properties of MC firing activity. However, the AHP characteristics can be directly altered by the membrane potential (present work), neuromodulation (Wu et al., 2002; Brosh et al., 2006), postnatal development (Duménieu et al., 2015; Yu et al., 2015), learning (Duménieu et al., 2015; Reuveni and Barkai, 2018), and recurrent synaptic transmission (Duménieu et al., 2015). Therefore, thanks to highly scalable characteristics, the AHP appears to be a key target for the modulation of olfactory bulb processing according to many parameters such as physiological state (e.g., reproduction period and food need), memory, and experience.

## Synthesis

Reviewing Editor: Matthew Grubb, King’s College London Decisions are customarily a result of the Reviewing Editor and the peer reviewers coming together and discussing their recommendations until a consensus is reached. When revisions are invited, a fact-based synthesis statement explaining their decision and outlining what is needed to prepare a revision will be listed below. The following reviewer(s) agreed to reveal their identity: Radha Kalluri, Lorcan Browne. Note: If this manuscript was transferred from JNeurosci and a decision was made to accept the manuscript without peer review, a brief statement to this effect will instead be what is listed below.

Reviewer 1:

The manuscript provides a nice analysis of dynamic fluctuations in voltage threshold to describe the bursting behavior of mitral cells. The combination of rigorous analysis of firing patterns, pharmacology, and modeling makes for an interesting paper highlighting the role of intrinsic biophysics in regulating the complex behavior of excitable cells. This version of the manuscript addresses many of my previous concerns related to focusing the presentation to make the manuscript more readable. I believe the authors have succeeded in doing this.

A few minor residual issues that the authors should consider addressing:

Introduction

- Consider splitting and organizing the introduction into multiple paragraphs.

- Is the buildup of a slow AHP component distinct from the accumulation of IA current or a manifestation of such? The discussion and data imply as much, but I’m reading this sentence as meaning that these are two different mechanisms leading to spike termination. “Balu and Strowbridge (2004) proposed that the burst termination was due to the build-up of the slow AHP during repetitive firing whereas a computational model suggested that burst termination would rely on the accumulation of potassium IA current during the firing (Rubin & Cleland, 2006).”

- “Here we provide evidences...” evidence not evidences.

- Consider spelling out OB rather using the abbreviation since it is used only a handful of times.

- Also consider replacing MP with Vm. I find it awkward to read MP when Vrest is used to refer to resting potential.

Results

- Figure 2B, label x-axis as Vrest

- Figure 4, all the symbols are very small and hard to see.

- “As expected, decreasing the density of Nav channels in the model, leaded to a shift of AP threshold toward more positive values (Fig 5B).” led not leaded. Here and elsewhere in the manuscript.

- “We showed that AHP acted as a regenerative mechanism that deinactivates Nav channels, leading to AP threshold negative shift and promoting firing of MCs in burst.” Awkward sentence in the transition to the slow AHP section.

- In contrast to the careful presentation of the results in the previous sections, the very last section of the results was poorly motivated and described.

- The description of the linear regression models used to predict AP thresholds, burst duration, and burst frequency was not sufficient for me to understand the implementation of the model. For example, are there systematic trends across cells such that some parameters are more highly weighted in all cells? I found the lack of description of the models to be especially problematic in the last section the results where the authors mention using a linear model to predict burst duration and frequency. As presented, the authors assume that the ready will readily appreciate the need for log() and loglog conversions etc., for the linear models to apply.

-

- “To further clarify the mechanisms of burst termination we used a linear model (using Vrest, AHP amplitude, AHP slope and AHP duration as parameters, see Methods for details) which predicts the dynamics of putative AP threshold after the last AP of the burst (see examples in Fig 8B, red dashed lines).”

Reviewer 2:

This study characterises, in some detail, the heterogeneity of firing in Mitral Cells (MC)s, focusing closely on the initiation, maintenance and termination of “bursts” of action potentials. By studying the preceding voltage trajectories and how they predict the properties of the following Action Potentials (APs), the authors neatly describe a mechanism for the apparent random onset of bursts, as well as the continued propagation of firing once an initial AP is generated. Broadly, this involving the dynamic movement of the AP threshold, as shifting membrane voltages makes more or fewer sodium channels available in the preceding timeframe. Finally, a mechanism is also offered for the cessation of these bursts, through the slow onset of an inactivating potassium conductance, which delays repolarisations and eventually bring the afterhyperpolarisation (AHP) rebound beneath the required AP threshold for further firing.

Unfortunately, as has been noted, some of the mechanistic detail is not wholly novel and rather acts to confirm much of our current understanding of neuronal firing and the role of AHP in dictating the firing properties of neuron. Nonetheless, the study is well designed, and the interpretation are justified based on the presented data. Whole cell patch-clamp experiments are well carried out, analysed in considerable detail and pharmacological and computational approaches nicely bolster their interpretations and conclusion.

It is worth acknowledging that the authors have made a considerable effort to address previous concerns about the presentation of data, streamlining the manuscript to focus on key results, reducing redundancy and aiding readability.

Whilst I believe the current manuscript would be a useful and interesting document to some in the field, I offer the following suggestions and concerns:

1.(Extended Data) Fig 7-1C & associated commentary in the text.

If I understand correctly, Fig 7-1C plots Relative AP threshold, a measure which uses Resting membrane potential in its derivation, against the actual Resting membrane potential itself. I do not understand the rational of this, correlating these two variables which will of course be highly co-dependent.

At present, it is not clear a claim such as “This effect was likely due to the stronger negative shift of AP threshold within the burst when the MP was depolarized (see Extended Data Fig 7-1C).” is justified, as slope of approx -1, reported here, is consistent with a model where AP threshold stayed relatively stable, but the Vm rest moving dynamically around this set point. i.e. 1mV decrease in Vm results in a 1 mV increase in Relative AP threshold, (presumably because Vm went down but AP threshold stayed the same).

Perhaps a plot of actual AP threshold against Resting membrane potential would be more informative?

2. Figure 6-4 (Extended data, and associated text in the main manuscript).

I am unconvinced by conclusions drawn from data presented in Figure 6-4. There is undoubtable an interesting question as to the relationship between OSNs input into MCs and the specific patterns of firing this might evoke, especially in the context of some of the result presented here. I understand these data were added in response to a previous enquiry as to the nature of MC firing in response to synaptic input. However, to begin to comment out the nature of this relationships, a deeper analysis of the inputs into MCs would need to be performed. The claim here, that spontaneous bursts of MC activity are “similar” to evoked ones, is based on too superficial an analysis to warrant any conclusions being drawn. Similarly, claims such as “... These results suggest that, once APs are generated, the bursting characteristics of MCs are determined by their intrinsic biophysical properties, i.e. the AHP, regardless the modality of AP induction.” have limited basis in data.

3.Figure 2B (left plot)

It is unclear the origin of this bimodal distribution of membrane potentials. Some reference is made to this in the figure legend, but a clearer description is needed of what these two groups of Vrest represent. Do they represent 2 populations of cells with different resting Membrane potentials? A small additional point: I’m not sure the mean and median make sense here when talking about a bimodal population.

4. Figure 7 in main text as well as Extended Figure 7-1, A-F

Membrane potential reported on the X-axis seems quite depolarised and doesn’t match the Membrane potentials reported in Figure 2B and elsewhere. Is Resting Membrane potential calculated differently here or is there some other explanation, i.e. Junction Potential not accounted for in these graphs?

5. Figure 5F Lower panels

The Y axis label “Na+ current variation” should be renamed “Na+ current change” or something similar. Variation carries connotations of variance or difference around a mean.

## Author Response

Dear Editor,

We have now completed the revision of our manuscript. All the remarks, questions and suggestions of reviewers have been taken into account. We have answered question by question in detail and we hope that our answers will satisfy their requirements.

We thank the referees for judging our work and allowing the improvement of our manuscript,

We very much hope that the current version of the manuscript can be published in eNeuro.

Best regards,

---------------------------------------------

Synthesis of Reviews:

Computational Neuroscience Model Code Accessibility Comments for Author:

Please ensure that the revised manuscript complies with eNeuro policy on computational neuroscience models:

Policy on Computer Code and Software

Computational models: New computational neuroscience computer code must be included with the initial submission as Extended Data to ensure reproducibility if published and be deposited in a suitable repository such as GitHub, ModelDB, BioModels, CellML, or Visiome. Studies using custom code deemed central to the conclusions must include a statement in the Materials and Methods section, under the heading “Code Accessibility", indicating whether and how the code can be accessed, including any accession numbers or restrictions and be cited in the references. The type of computer and the operating system used to run the code in the manuscript must be included in the Materials and Methods section. Code must be available upon acceptance and publication of the manuscript. No code is to be withdrawn following publication. Code files must be packaged in a single ZIP file.

Reviewer 1:

The manuscript provides a nice analysis of dynamic fluctuations in voltage threshold to describe the bursting behavior of mitral cells. The combination of rigorous analysis of firing patterns, pharmacology, and modeling makes for an interesting paper highlighting the role of intrinsic biophysics in regulating the complex behavior of excitable cells. This version of the manuscript addresses many of my previous concerns related to focusing the presentation to make the manuscript more readable. I believe the authors have succeeded in doing this.

A few minor residual issues that the authors should consider addressing:

Introduction

- Consider splitting and organizing the introduction into multiple paragraphs.

The introduction has been split into 3 paragraphs to better guide the reader.

- Is the buildup of a slow AHP component distinct from the accumulation of IA current or a manifestation of such? The discussion and data imply as much, but I’m reading this sentence as meaning that these are two different mechanisms leading to spike termination. “Balu and Strowbridge (2004) proposed that the burst termination was due to the build-up of the slow AHP during repetitive firing whereas a computational model suggested that burst termination would rely on the accumulation of potassium IA current during the firing (Rubin & Cleland, 2006).”

In fact, in our study the buildup of slow AHP and the accumulation of IA current are confounded. However, IA is not an AHP current per se, since it is deinactivated by AHP itself (IA is deinactivated by hyperpolarisation). The accumulation of IA is rather a consequence of AHP. This explains the distinction made in the sentence quoted by the reviewer. We have reworded the sentence to make this clearer with: “A computational model refined this idea by proposing that burst termination is not due to the accumulation of classical AHP currents but rather by the progressive deinactivation of potassium IA current along the consecutive AHPs of the burst (Rubin & Cleland, 2006).”

- “Here we provide evidences...” evidence not evidences.

This has been corrected

- Consider spelling out OB rather using the abbreviation since it is used only a handful of times.

The 3 occurrences of OB have been spelled out.

- Also consider replacing MP with Vm. I find it awkward to read MP when Vrest is used to refer to resting potential.

We acknowledged the reviewer concern and replaced MP by Vm in text and figures.

Results

- Figure 2B, label x-axis as Vrest

This has been done

- Figure 4, all the symbols are very small and hard to see.

Symbol size has been increased

- “As expected, decreasing the density of Nav channels in the model, leaded to a shift of AP threshold toward more positive values (Fig 5B).” led not leaded. Here and elsewhere in the manuscript.

All occurrences of “leaded” have been corrected by “led"

- “We showed that AHP acted as a regenerative mechanism that deinactivates Nav channels, leading to AP threshold negative shift and promoting firing of MCs in burst.” Awkward sentence in the transition to the slow AHP section.

Of course this sentence was awkward, thus we removed it

- In contrast to the careful presentation of the results in the previous sections, the very last section of the results was poorly motivated and described.

In addition to a better model description (see below), we have slightly reworded the last result section content in order to better describe our motivation and analysis

- The description of the linear regression models used to predict AP thresholds, burst duration, and burst frequency was not sufficient for me to understand the implementation of the model. For example, are there systematic trends across cells such that some parameters are more highly weighted in all cells? I found the lack of description of the models to be especially problematic in the last section the results where the authors mention using a linear model to predict burst duration and frequency. As presented, the authors assume that the ready will readily appreciate the need for log() and loglog conversions etc., for the linear models to apply.

We have taken into account the reviewer concerns about the description of linear models in particular in the last section of the results. Consequently, we added a new “methods” section specifically describing the linear models used in the last result section. In particular the new “methods” section motivates the use of log() and log(log()) transformation of the endogenous variables in order to have normality and heteroscedasticity of the residuals. The models of this section pooled data from all cells, thus no information about individual cell is available. This is now clearly stated in the methods and results sections

For the threshold models, a model was fitted for each cell but we did not compared results across cells because it would have been redundant with our analysis of each parameter done individually in Figure 3, 6 and 7.

-

- “To further clarify the mechanisms of burst termination we used a linear model (using Vrest, AHP amplitude, AHP slope and AHP duration as parameters, see Methods for details) which predicts the dynamics of putative AP threshold after the last AP of the burst (see examples in Fig 8B, red dashed lines).”

This reviewer comment seems incomplete (unless it is related to the previous one) and we found nothing wrong with it

Reviewer 2:

This study characterises, in some detail, the heterogeneity of firing in Mitral Cells (MC)s, focusing closely on the initiation, maintenance and termination of “bursts” of action potentials. By studying the preceding voltage trajectories and how they predict the properties of the following Action Potentials (APs), the authors neatly describe a mechanism for the apparent random onset of bursts, as well as the continued propagation of firing once an initial AP is generated. Broadly, this involving the dynamic movement of the AP threshold, as shifting membrane voltages makes more or fewer sodium channels available in the preceding timeframe. Finally, a mechanism is also offered for the cessation of these bursts, through the slow onset of an inactivating potassium conductance, which delays repolarisations and eventually bring the afterhyperpolarisation (AHP) rebound beneath the required AP threshold for further firing.

Unfortunately, as has been noted, some of the mechanistic detail is not wholly novel and rather acts to confirm much of our current understanding of neuronal firing and the role of AHP in dictating the firing properties of neuron. Nonetheless, the study is well designed, and the interpretation are justified based on the presented data. Whole cell patch-clamp experiments are well carried out, analysed in considerable detail and pharmacological and computational approaches nicely bolster their interpretations and conclusion.

It is worth acknowledging that the authors have made a considerable effort to address previous concerns about the presentation of data, streamlining the manuscript to focus on key results, reducing redundancy and aiding readability.

Whilst I believe the current manuscript would be a useful and interesting document to some in the field, I offer the following suggestions and concerns:

1.(Extended Data) Fig 7-1C & associated commentary in the text.

If I understand correctly, Fig 7-1C plots Relative AP threshold, a measure which uses Resting membrane potential in its derivation, against the actual Resting membrane potential itself. I do not understand the rational of this, correlating these two variables which will of course be highly co-dependent.

At present, it is not clear a claim such as “This effect was likely due to the stronger negative shift of AP threshold within the burst when the MP was depolarized (see Extended Data Fig 7-1C).” is justified, as slope of approx -1, reported here, is consistent with a model where AP threshold stayed relatively stable, but the Vm rest moving dynamically around this set point. i.e. 1mV decrease in Vm results in a 1 mV increase in Relative AP threshold, (presumably because Vm went down but AP threshold stayed the same).

Perhaps a plot of actual AP threshold against Resting membrane potential would be more informative?

Actually there is no a priori reason why Vrest and the relative AP threshold should be correlated. Indeed, the relative AP threshold is the difference of two independent variables (the actual AP threshold and Vrest) and thus may mathematically be completely uncorrelated with Vrest.

However, as shown in our manuscript, when Vrest increases we have 3 simultaneous effects that tend to decrease the relative AP threshold: AHP amplitude decreases (meaning larger hyperpolarization relative to Vrest) but slower than the increase of Vrest, the AHP slope increases, and even if the AHP amplitude remained stable, the decrease of Nav availability at higher Vrest would increase the effect of the AHP on following AP thresholds (see model in Fig 7-1H). Overall, it seems fortuitous to us that the correlation with Vrest is on average -1, which means the actual AP threshold within burst is on average constant when Vrest is increased.

Further confirmation of this coincidence is that when, instead of examining within cell data, we averaged the cell measurements and then examined the population level, all correlations are qualitatively the same, but quantitatively, the slope of correlation between the relative AP threshold and Vrest is now -0.2 .

2. Figure 6-4 (Extended data, and associated text in the main manuscript).

I am unconvinced by conclusions drawn from data presented in Figure 6-4. There is undoubtable an interesting question as to the relationship between OSNs input into MCs and the specific patterns of firing this might evoke, especially in the context of some of the result presented here. I understand these data were added in response to a previous enquiry as to the nature of MC firing in response to synaptic input. However, to begin to comment out the nature of this relationships, a deeper analysis of the inputs into MCs would need to be performed. The claim here, that spontaneous bursts of MC activity are “similar” to evoked ones, is based on too superficial an analysis to warrant any conclusions being drawn. Similarly, claims such as “... These results suggest that, once APs are generated, the bursting characteristics of MCs are determined by their intrinsic biophysical properties, i.e. the AHP, regardless the modality of AP induction.” have limited basis in data.

Indeed, we agreed that due to the limited number of recorded neurons the data are not sufficient to draw definitive conclusions. In our experimental conditions the probability to record evoked activity in slice was quite low. Thus, recording a sufficient number of stimulated neurons under these conditions to be able to perform a complete analysis of the question is a task in itself that cannot be performed in the context of the present work. Nevertheless, we think these data remained interesting and we have reworded the paragraph in the results section and the supplementary figure legend to emphasize their nature of preliminary analysis.

3.Figure 2B (left plot)

It is unclear the origin of this bimodal distribution of membrane potentials. Some reference is made to this in the figure legend, but a clearer description is needed of what these two groups of Vrest represent. Do they represent 2 populations of cells with different resting Membrane potentials? A small additional point: I’m not sure the mean and median make sense here when talking about a bimodal population.

The bimodality of Vrest distribution is due to intrinsic differences of Vrest between cells (15 cells around -55mV, and 17 around -60mV), while also amplified by the fact that some cells did not have spontaneous activity at Vrest and were slightly depolarized (15 cells depolarized around -55mV); by the way, 2 cells needed to be hyperpolarized around -60mV in order to prevent continuous activity. Overall burst activity was observed either at Vrest or after a small current injection. Since we did not observe any difference between the two groups of cells in our analyses, the groups were pooled. This is now clearly stated in the methods and results sections.

4. Figure 7 in main text as well as Extended Figure 7-1, A-F

Membrane potential reported on the X-axis seems quite depolarised and doesn’t match the Membrane potentials reported in Figure 2B and elsewhere. Is Resting Membrane potential calculated differently here or is there some other explanation, i.e. Junction Potential not accounted for in these graphs?

Indeed, we forgot to take into account the junction potential in these figures, it is now corrected.

5. Figure 5F Lower panels

The Y axis label “Na+ current variation” should be renamed “Na+ current change” or something similar. Variation carries connotations of variance or difference around a mean.

The proposed change has been made
