# Supplementary figures and images for: Afterhyperpolarization Promotes the Firing of Mitral Cells through a Voltage-Dependent Modification of Action Potential Threshold
Source: eNeuro. 2022 Apr 1;9(2):ENEURO.0401-21.2021. doi: 10.1523/ENEURO.0401-21.2021 (PMC8982644; doi:10.1523/ENEURO.0401-21.2021)

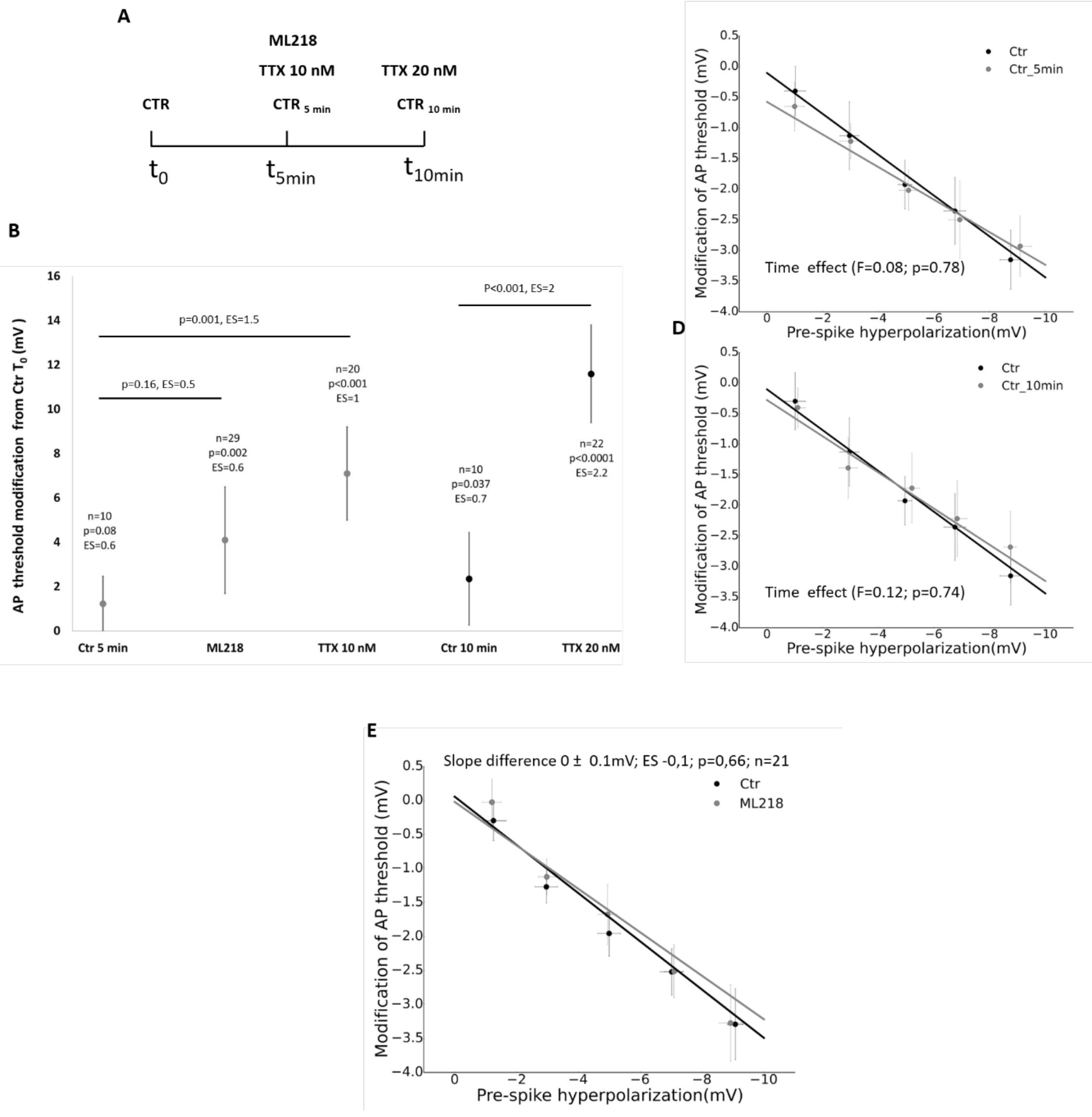

Supplement: Figure 5-1 — Modification of MC AP threshold and hyperpolarization effects over time and after applications of Na+ and Ca2+ channels antagonists. A, Timing of experiments during which AP threshold was measured after 5 and 10 min, in control conditions or after different pharmacological applications. B, AP threshold was significantly increased by blockade of Na+ channels with TTX, but not by the antagonist of T-type Ca2+ channels (ML218; 5–10 μm). Note the spontaneous increase of threshold with time in the control condition, both at 5 and 10 min, with a small difference, possibly due to cell dialysis. The statistical analysis compared with Ctrt0 is depicted above the data points. Values are expressed as the modification of AP threshold compared with the control condition at time 0. C, D, The effect of membrane hyperpolarization on the AP threshold was stable with time. E, The antagonist of T-type channels ML218 did not affect the modification of AP threshold produced by membrane hyperpolarization. Horizontal and vertical bars represent 95% confidence interval. Download Figure 5-1, TIF file. [file enu-eN-NWR-0401-21-s01.tif]

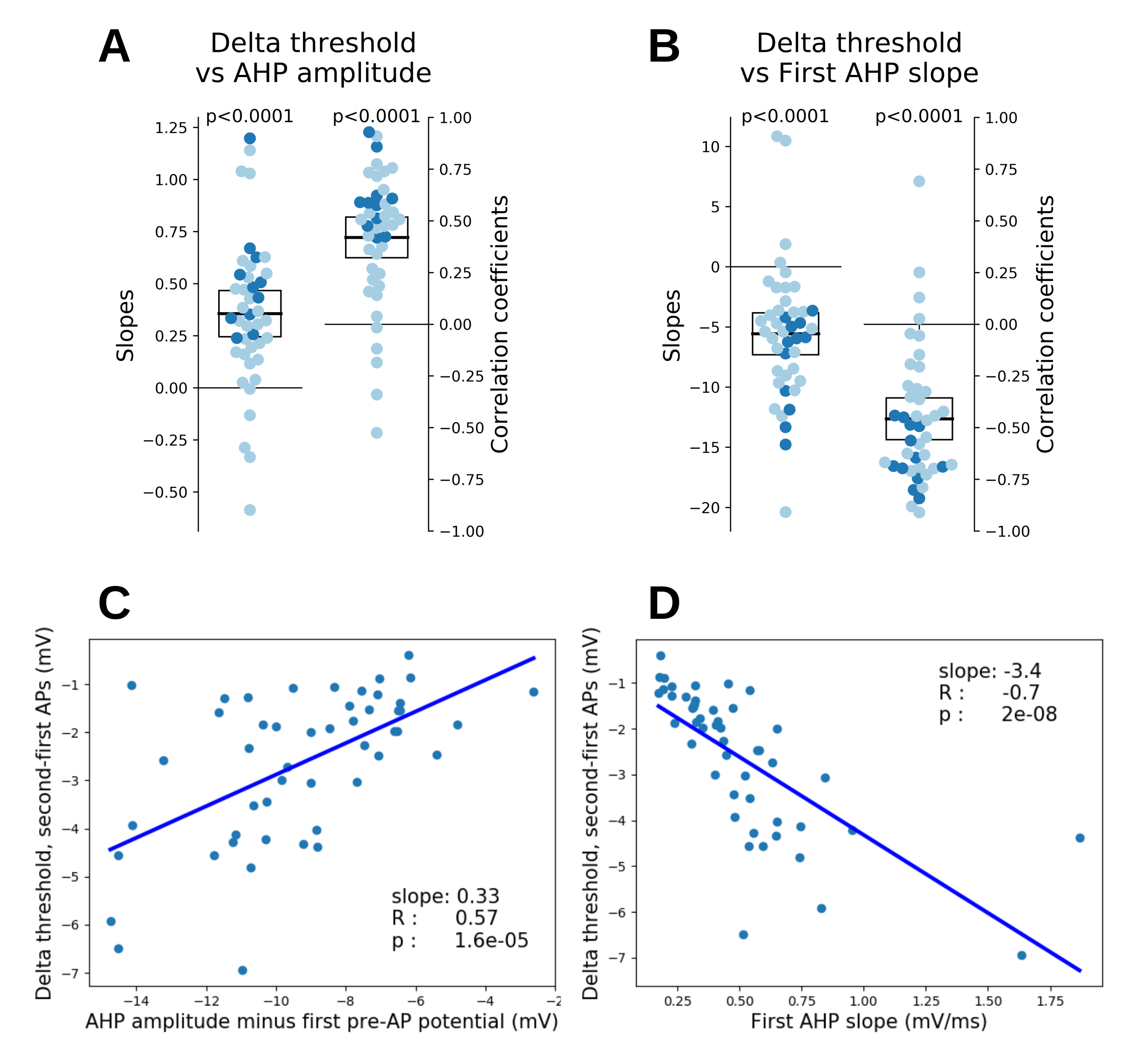

Supplement: Figure 6-1 — Characteristics of the first AHP determined the negative shift of AP threshold between the first AP and second AP of the burst. A, Distributions of slopes and correlation coefficients of the linear correlations performed in Figure 6C, left, of the main text (slope, 0.35 ± 0.10 mV/mV; ES = 1.03; R, 0.42 ± 0.09; t test, t = 9.34; p < 0.0001; N = 49). Darker dots correspond to individual fits with p < 0.05 (Pearson correlation, corrected for multiple comparisons). B, Distributions of slopes and correlation coefficients of the linear correlations performed in Figure 6C, right, of the main text [slope, –5.6 ± 1.5 mV/(mV/ms); ES = –1.04; R, –0.47 ± 0.09; t test, t = –10.4; p < 0.0001; N = 49]. Darker dots correspond to individual fits with p < 0.05 (Pearson correlation, corrected for multiple comparisons). C, Linear correlation between average first AHP amplitude and average negative shift of AP threshold (slope, 0.33 mV/mV; R = 0.57; Wald test, p < 0.001; N = 49). Each dot represents the average values for a given cell. D, Linear correlation between average first AHP slope and average negative shift of AP threshold [slope, –3.4 mV/(mV/ms); R = –0.7; Wald test, p < 0.001; N = 49]. Each dot represents the average values for a given cell. Download Figure 6-1, TIF file. [file enu-eN-NWR-0401-21-s02.tif]

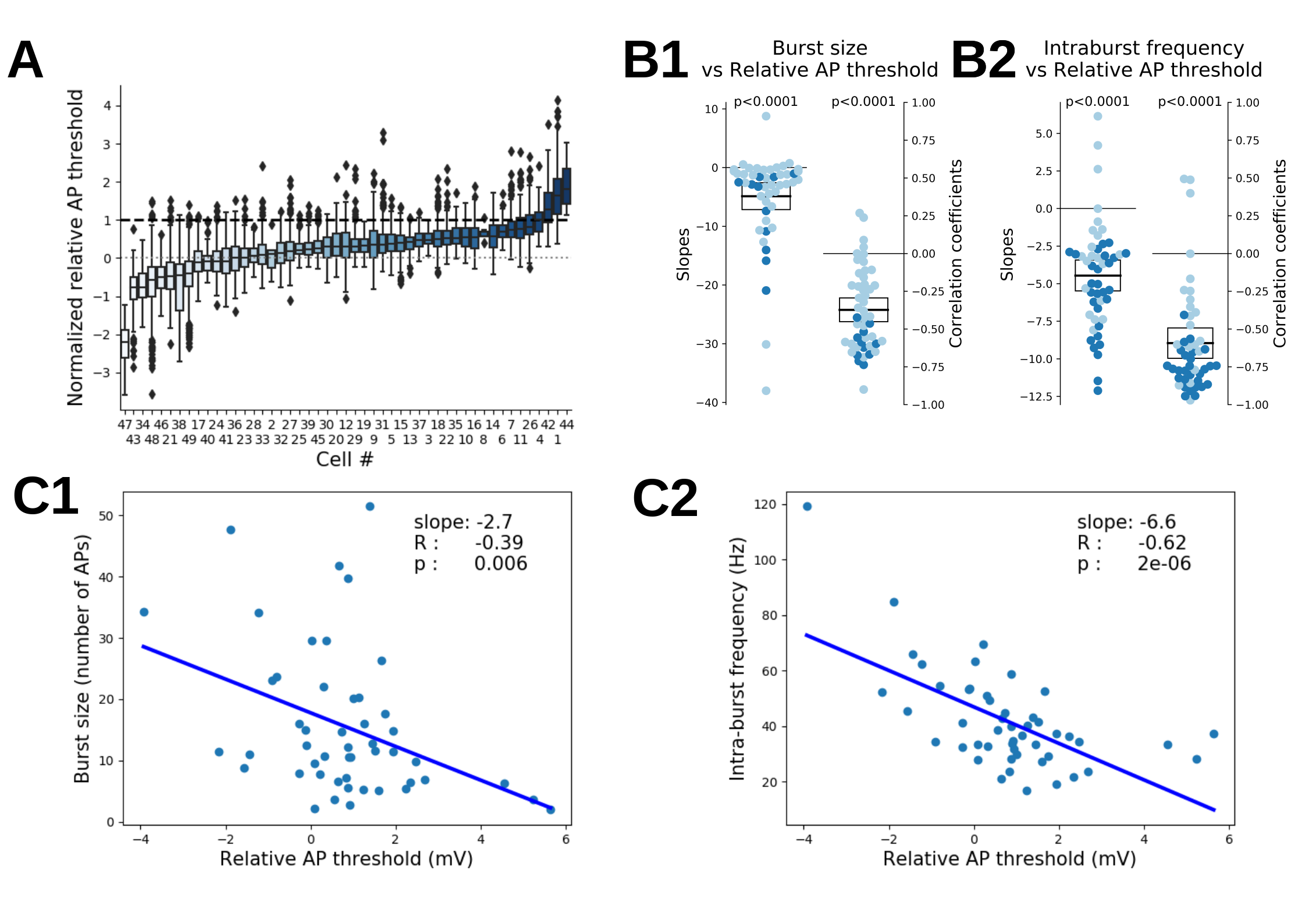

Supplement: Figure 6-2 — Influence of intraburst relative AP threshold on burst size (number of APs) and intraburst AP frequency. A, Same analysis as in Figure 6D of the main text but for each MC. B1, Distributions of slopes and correlation coefficients of the linear correlations performed in Figure 6E, left, of the main manuscript (slope, –4.87 ± 2.22 AP/mV; ES = –0.62; R, –0.37 ± 0.08; t test, t = –9.56; p < 0.0001; N =49). Darker dots correspond to individual fits with p < 0.05 (Pearson correlation, corrected for multiple comparisons). B2, Distributions of slopes and correlation coefficients of the linear correlations performed in Figure 6E, right, of the main manuscript (slope, –4.46 ± 1.00 Hz/mV; ES = –1.26; R, –0.59 ± 0.10; t test, t = –11.9; p < 0.0001; N = 49). Correlation between the intraburst frequency and relative AP threshold: individual-cell fit results from data shown in Figure 5C, right: average slope = – 4.46 ± 1.00 Hz/mV; ES = –1.26; average R = –0.59 ± 0.10; t test, t = –11.9; p < 0.0001; N = 49. Darker dots correspond to individual fits with p < 0.05 (Pearson correlation, corrected for multiple comparisons). C1, Linear correlation between average relative AP threshold and average burst size (R, correlation coefficient; p, Wald test p-value; N = 49). Each dot represents the average values for a given cell. C2, Linear correlation between average relative AP threshold and average intraburst frequency (R, correlation coefficient; p, Wald test p-value; N = 49). Each dot represents the average values for a given cell. Download Figure 6-2, TIF file. [file enu-eN-NWR-0401-21-s03.tif]

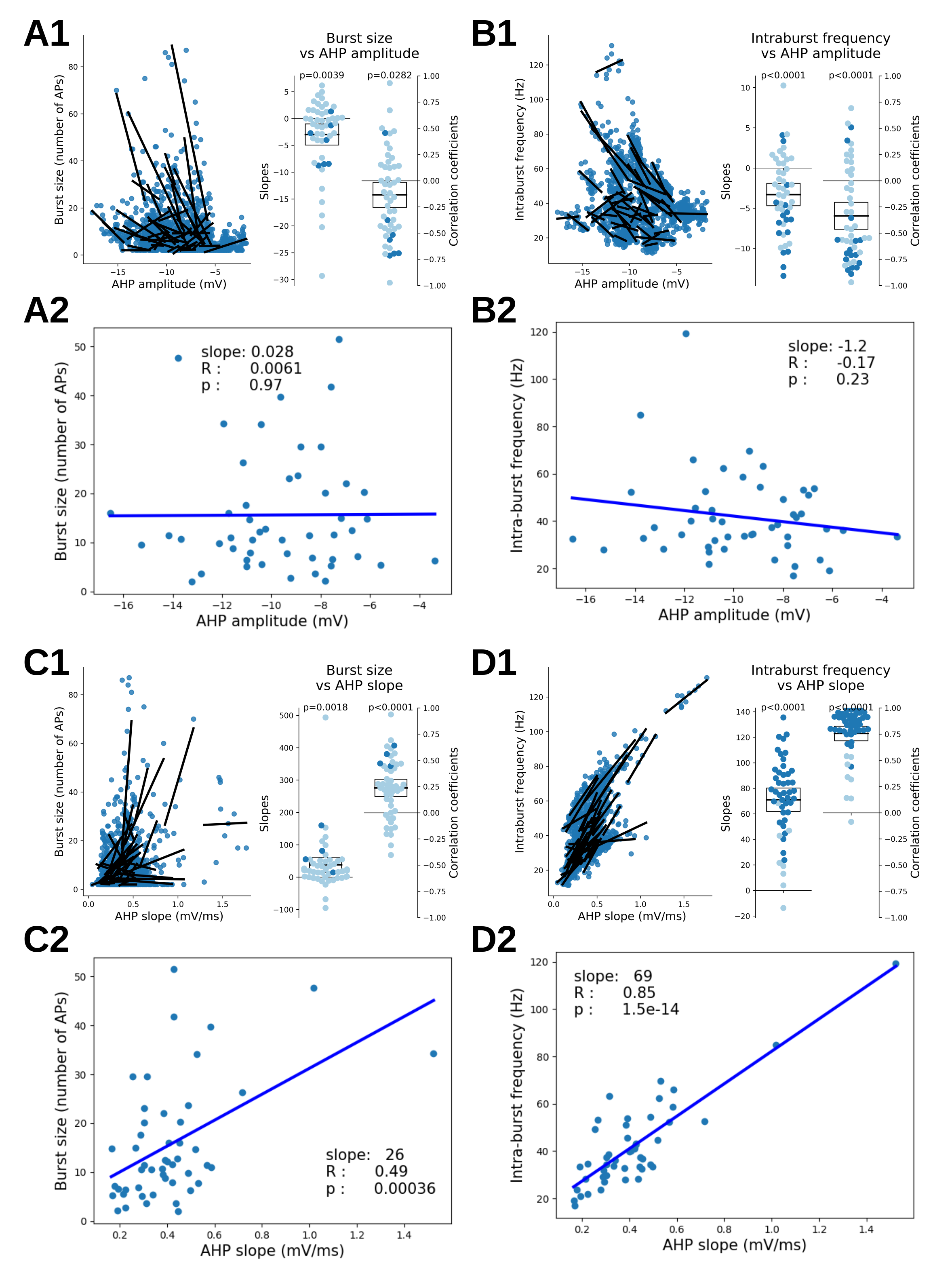

Supplement: Figure 6-3 — AHP characteristics determined the firing properties of bursts, namely the burst size and intraburst AP frequency. A, Larger AHP amplitudes were associated with longer bursts. A1, Left, Correlations between AHP amplitude and burst size are found in a cell per cell analysis. Right, Distributions of slopes and correlation coefficients (slope, –2.98 ± 1.92 AP/mV; ES = –0.43; R, –0.13 ± 0.12; t test t = –2.26; p = 0.03; N = 49). Darker dots correspond to individual fits with p < 0.05 (Pearson correlation, corrected for multiple comparisons). A2, Linear correlation between average AHP amplitude and average burst size (R, correlation coefficient; p, Wald test p-value; N = 49). Each dot represents the average values for a given cell. B, Larger AHP amplitudes were associated with higher intraburst frequency. B1, Left, Correlations between AHP amplitude and intraburst frequency is found in a cell per cell analysis. Right, Distributions of slopes and correlation coefficients (slope, –3.31 ± 1.36 Hz/mV; ES = –0.69; R, –0.33 ± 0.12; t test, t = –5.23; p < 0.0001; N = 49). Darker dots correspond to individual fits with p < 0.05 (Pearson correlation, corrected for multiple comparisons). B2, Linear correlation between average AHP amplitude and average intraburst frequency (R, correlation coefficient; p, Wald test; N = 49). Each dot represents the average values for a given cell. C, Faster AHP repolarizations (i.e., larger AHP slopes) were associated with longer bursts. C1, Left, Correlations between AHP slope and burst size are found in a cell per cell analysis. Right, Distributions of slopes and correlation coefficients [slope, 38.3 ± 22.7 AP/(mV/ms); ES = 0.48; p = 0.002; R = 0.24 ± 0.08; t test, t = 5.79; p < 0.0001; N = 49). Darker dots correspond to individual fits with p < 0.05 (Pearson correlation, corrected for multiple comparisons). C2, Linear correlation between average AHP slope average and burst size (R, correlation coefficient; p, Wald test p-value; N = 49). D, Faster [file enu-eN-NWR-0401-21-s04.tif]

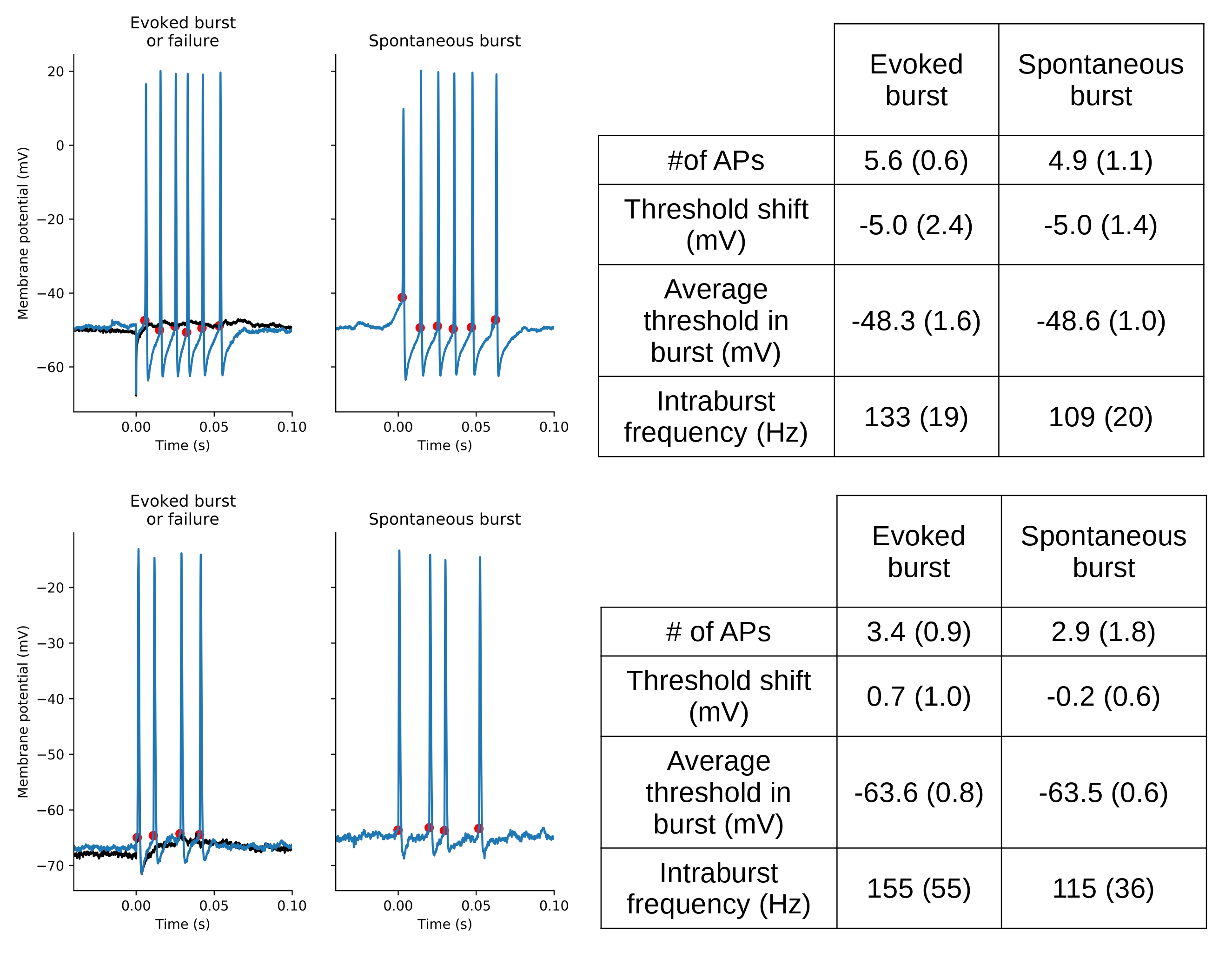

Supplement: Figure 6-4 — Synaptically evoked bursts are similar to spontaneous bursts. Examples of a cell that displayed a clear threshold shift between the first two APs (top panels) and another that did not (bottom panels). For each cell, left panels illustrate side by side a burst evoked by olfactory nerve stimulation and a spontaneous burst. Black lines, An example of stimulation that failed to evoke a burst; red dots, represent AP thresholds. Right tables compare features of evoked and spontaneous bursts (for top cell: 3 evoked bursts and 21 spontaneous bursts; for bottom cell: 5 evoked bursts and 367 spontaneous bursts). Table values are displayed as the mean (SD). Download Figure 6-4, TIF file. [file enu-eN-NWR-0401-21-s06.tif]

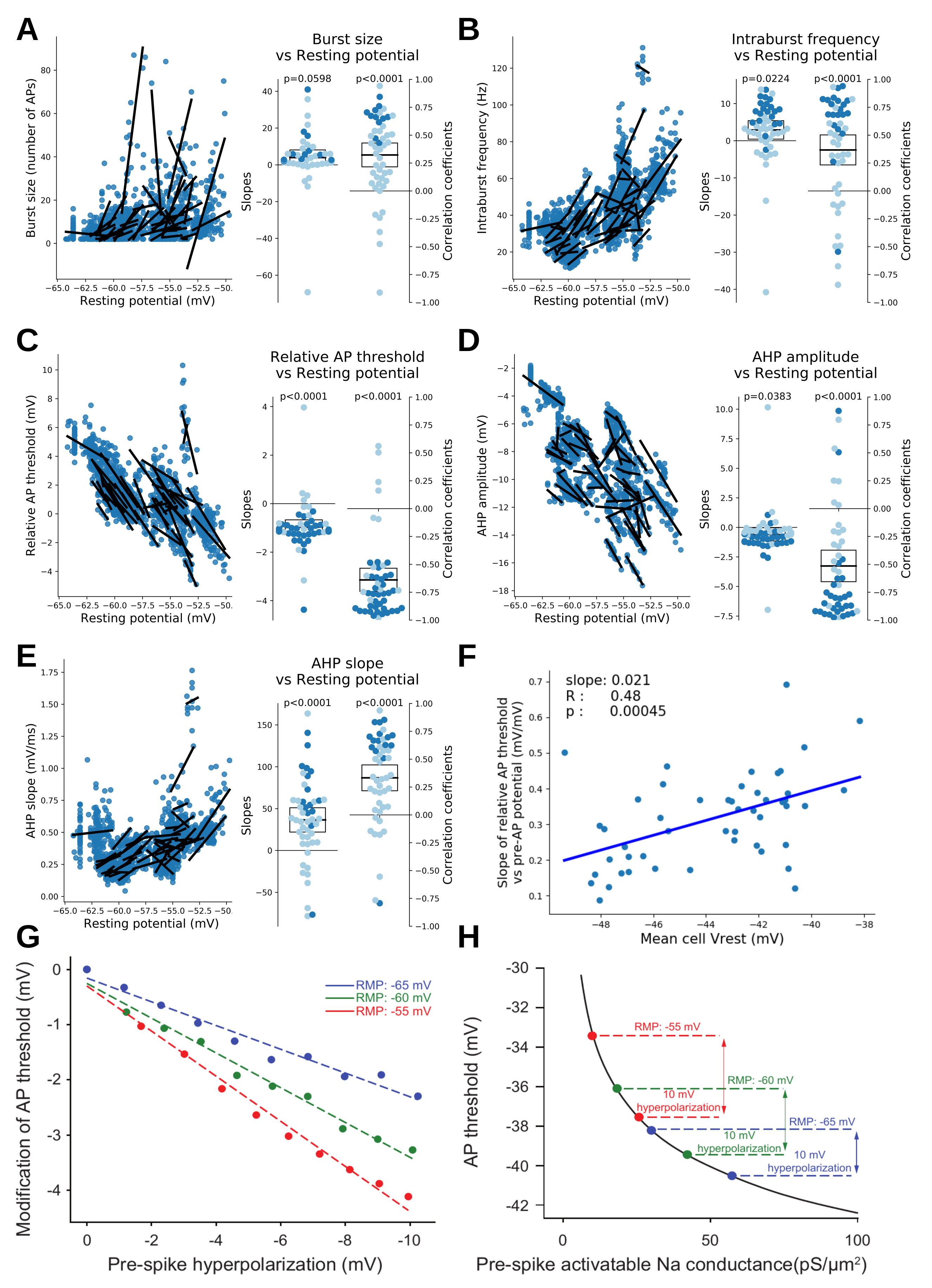

Supplement: Figure 7-1 — Influence of resting potential on burst size and intraburst frequency was linked to the modification of AP threshold, through changes in AHP characteristics and sodium channels inactivation rates. A–E, Linear fits, slopes, and coefficients of the correlations between resting potential before burst and burst firing properties, AP threshold characteristics, or AHP characteristics. The first AP threshold in the burst is not taken into account. The results are presented as in Figure 3C (values are averaged per burst, and fits are done within each cell). A, Burst size increased with Vm depolarization (slope, 3.95 ± 4.01 AP/mV; ES = 0.28; R, 0.32 ± 0.10; t test, t = 6.06; p < 0.0001; N = 49; left, identical to Fig. 7A). B, Intraburst frequency increased with Vm depolarization (slope, 2.90 ± 2.41 Hz/mV; ES = 0.34; R, 0.37 ± 0.13; t test, t = 5.49, p < 0.0001; N = 49; left, identical to Fig. 7B). C, Relative AP threshold became more negative with Vm depolarization (slope, –0.97 ± 0.29 mV/mV; ES = –0.94; R, –0.64 ± 0.10; t test, t = –12.3; p < 0.0001; N = 49). D, AHP amplitude increased with Vm depolarization (slope, –0.59 ± 0.55 Hz/mV; ES = –0.30; R, –0.51 ± 0.14; t test, t = –7.32; p < 0.0001, N = 49). E, AHP repolarization speed increased with Vm depolarization [slope, 0.036 ± 0.014 (mV/ms)/mV; ES = 0.73; average R, 0.33 ± 0.11; t test, t = 5.75; p < 0.0001; N = 49]. F, Cells with a more depolarized Vrest showed a greater effect of pre-AP potential on AP threshold. In this panel, all the spikes of the burst are analyzed. For the first spike, pre-AP potential is the most hyperpolarized value of membrane potential preceding the burst while for the other APs, it corresponds to the negative peak of the preceding AHP (Fig. 1). Note that slopes of the correlation between relative AP threshold and pre-AP potential were bigger at more depolarized Vrest (i.e, the effect of a given hyperpolarization on AP threshold is bigger when resting potential is more depolarized). G, Simulatio [file enu-eN-NWR-0401-21-s05.tif]

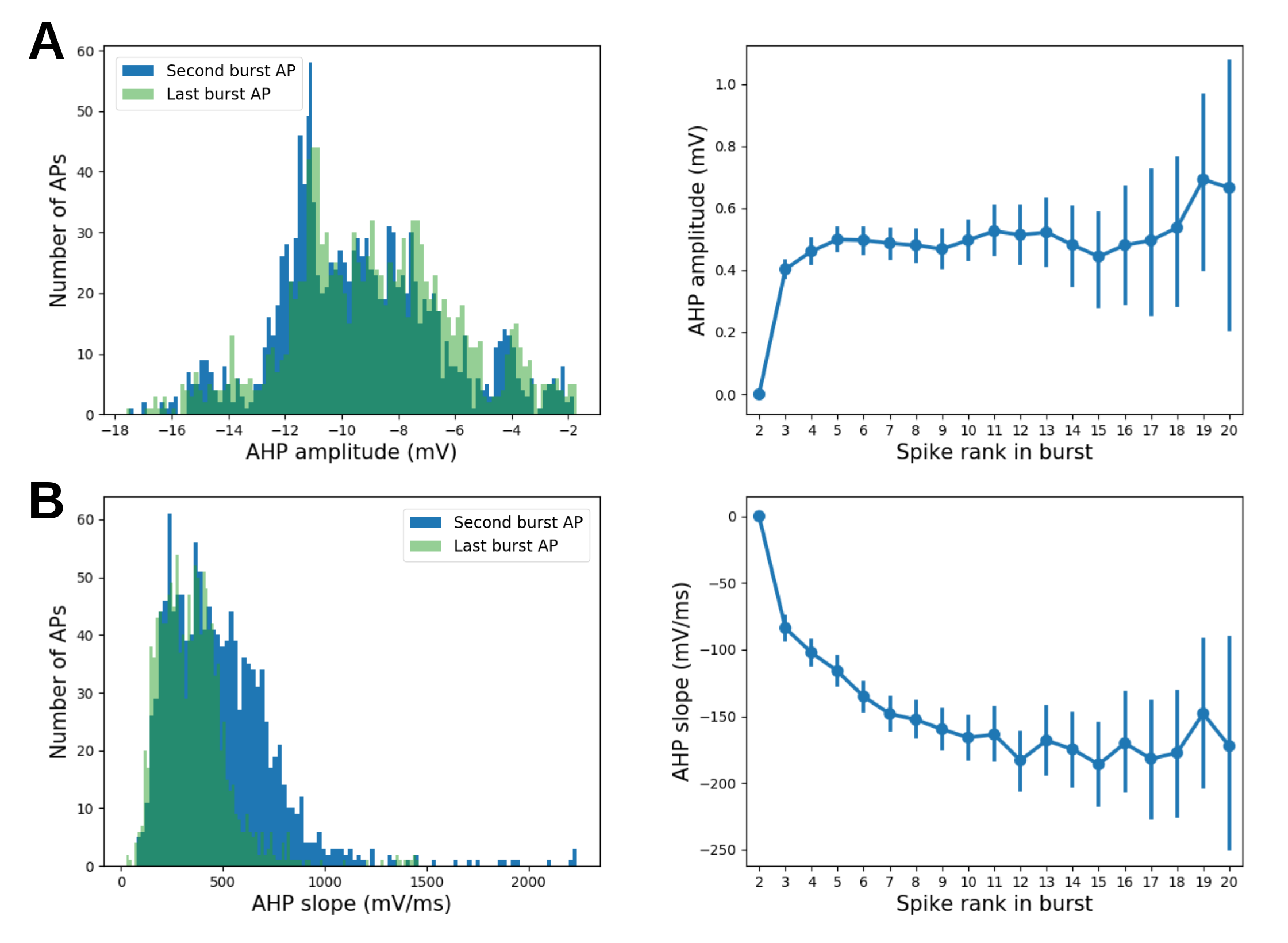

Supplement: Figure 8-1 — Evolution of AHP parameters during bursts. A, Same graphs as in Figure 8A (main text), but for AHP amplitude preceding the AP. Left, Distributions of AHP amplitude before the second AP and the last AP of the burst. There was a small decrease of AHP amplitude from the second to last spike (0.46 ± 0.04 mV; paired t test, t = 23.5; p < 0.001; N = 1276; ES = 0.65). Right, A small decrease of absolute AHP amplitude from the second to the third AP in burst, but after the third AP in burst the average AHP amplitude was constant. B, Same graphs as in Figure 8A (main text), but for AHP slope preceding the AP. Left, Distributions of AHP slope before the second AP and the last AP of the burst. There was a decrease of AHP slope from second to last AP (–132 ± 9 mV/ms; paired t test, t = –27.8; p < 0.001; N = 1276; ES = –0.77). Right, The decrease of AHP slope occurs mainly during the first 10 APs in burst. Download Figure 8-1, TIF file. [file enu-eN-NWR-0401-21-s07.tif]
